# HIGH RATES OF EVOLUTION PRECEDED THE ORIGIN OF BIRDS

**DOI:** 10.1111/evo.12363

**Published:** 2014-02-23

**Authors:** Mark N Puttick, Gavin H Thomas, Michael J Benton, P David Polly

**Affiliations:** 1 School of Earth Sciences, University of BristolWills Memorial Building, Queen's Road, Bristol, BS8 1RJ, United Kingdom; 3 Department of Animal and Plant Sciences, University of SheffieldAlfred Denny Building, Western Bank, Sheffield S10 2TN, United Kingdom

**Keywords:** Aves, birds, dinosaurs, evolution, flight, morphology

## Abstract

The origin of birds (Aves) is one of the great evolutionary transitions. Fossils show that many unique morphological features of modern birds, such as feathers, reduction in body size, and the semilunate carpal, long preceded the origin of clade Aves, but some may be unique to Aves, such as relative elongation of the forelimb. We study the evolution of body size and forelimb length across the phylogeny of coelurosaurian theropods and Mesozoic Aves. Using recently developed phylogenetic comparative methods, we find an increase in rates of body size and body size dependent forelimb evolution leading to small body size relative to forelimb length in Paraves, the wider clade comprising Aves and Deinonychosauria. The high evolutionary rates arose primarily from a reduction in body size, as there were no increased rates of forelimb evolution. In line with a recent study, we find evidence that Aves appear to have a unique relationship between body size and forelimb dimensions. Traits associated with Aves evolved before their origin, at high rates, and support the notion that numerous lineages of paravians were experimenting with different modes of flight through the Late Jurassic and Early Cretaceous.

Did preadaptations for flight precede the origin of birds (Aves)? The origin of flight in birds is one of the great evolutionary transitions and has received considerable attention in recent years (Padian and Chiappe 1998; Clarke and Middleton 2008; Dececchi and Larrson 2009; Benson and Choiniere 2013; Dececchi and Larrson 2013). The evolution of birds is often considered coincident with the origins of flight, but many traits associated with flight evolved before the origin of Aves (Padian and Chiappe 1998). Furthermore, the discovery of new fossils, especially from China (Hu *et al.* 2009; Xu *et al.* 2011, 2003; Godefroit *et al.* 2013; Zheng *et al.* 2013), and the publication of new phylogenies (Turner *et al.* 2012; Godefroit *et al.* 2013; O'Connor and Zhou 2013) and functional studies (Gatesy 1990; Gatesy and Dial 1996; Middleton and Gatesy 2000; Turner *et al.* 2007; Clarke and Middleton 2008; Nesbitt *et al.* 2009; Nudds and Dyke 2010; Sullivan *et al.* 2010; Novas *et al.* 2012; Allen *et al.* 2013; Dececchi and Larrson 2013) continue to challenge and shape our understanding of the route to, and appearance of, the morphological conditions required for flight. What was once seen as a rapid adaptive radiation, in which *Archaeopteryx* rapidly acquired 30 or more avian apomorphies, is now seen as a stepwise process of more than 50 million years (Padian and Chiappe 1998). Different names have been applied to birds, with Aves sometimes restricted to the crown group; but here we use Aves in the traditional sense, to refer to the clade that encompasses *Archaeopteryx*, all extant birds, and all the fossil forms in between (Padian and Chiappe 1998).

Large-scale events and patterns in macroevolution may reveal a great deal of information about the acquisition of novel characters when they are assessed using new numerical methods. Here, we study the origin of birds (Aves) from bird-line archosaurs (theropod dinosaurs) using recently developed phylogenetic comparative methods (PCMs). PCMs have frequently been used in biology to investigate rates and modes of evolution using molecular phylogenies (O'Meara *et al.* 2006; Thomas *et al.* 2009; Harmon *et al.* 2010; Venditti *et al.* 2011) and are increasingly being employed to complement classical numerical techniques in paleobiology (Sookias *et al.* 2012; Benson and Choiniere 2013; Zanno and Mackovicky 2013).

“Avian” traits are a mixture of Aves-specific characters, and those shared with the wider clade of related theropod dinosaurs (Padian and Chiappe 1998). Many morphological changes associated with Aves are now known to have predated the origin of the birds, and have origins unrelated to flight (Padian and Chiappe 1998; Sullivan *et al.* 2010). There is disagreement, however, about the relative timing of two important flight-related adaptations, body size miniaturization and forelimb lengthening. Many authors place the reduction in body size on the line to Paraves, the clade comprising Aves and Deinonychosauria (Xu *et al.* 2003; Turner *et al.* 2007; Novas *et al.* 2012). However, a recent study investigating rates of morphological change using PCM (Benson and Choiniere 2013) showed increased rates of hindlimb evolution in Pygostylia, a clade nested within Aves, and although it was not explicitly a study of body-size evolution, it did not detect miniaturization of the Paraves. A further substantial change associated with flight was elongation of the forelimb relative to body size, which has been found to be exclusive to Aves (Dececchi and Larrson 2013). It remains unclear whether these are Aves-specific changes (Clarke and Middleton 2008; Dececchi and Larrson 2013), or changes that began prior to, and continued after, the origin of Aves (Dececchi and Larrson 2009; Novas *et al.* 2012).

Here, we attempt to resolve the controversies over the origins of avian flight using a series of phylogenetic comparative analyses of the trajectories and rates of evolution of body size and forelimb length. Rates of character trait evolution are of huge importance in biology, and particularly in paleobiology (Gingerich 2009). Rates of phenotypic evolution in the fossil record can be measured using both phylogenetic (Eastman *et al.* 2011; Venditti *et al.* 2011; Thomas and Freckleton 2012; Slater 2013) and nonphylogenetic approaches (Hunt 2007). Evolutionary rates can vary among the branches of phylogenetic trees in subtle ways that can be difficult to disentangle but can have different evolutionary interpretations. Of particular relevance is whether miniaturization and forelimb elongation (1) evolved rapidly preceding Aves, (2) evolved rapidly preceding Paraves, (3) was a rapid but gradual process nested within either of these clades, or (4) followed a hitherto unexpected pattern.

We find that, as noted before (Padian and Chiappe 1998; Sullivan *et al.* 2010), morphological changes associated with flight preceded the origin of Aves. Our results largely support those previous analyses of character evolution in bird-line archosaurs that infer miniaturization leading to the Paraves (Turner *et al.* 2007; Novas *et al.* 2012), with no apparent changes in rates of forelimb evolution (Dececchi and Larrson 2013), leading to a larger forelimb for a given body size in the Paraves. Additionally, we observe that the relationship between forelimb length and body size varies between nonparavian theropods, Paraves, and Aves (Dececchi and Larrson 2013), but the precise interpretation differs between different phylogenies and taxonomic definitions. Overall, our results consistently show a move to a small body size, without a concurrent reduction in forelimb length, in Paraves.

## Materials and Methods

### PHYLOGENIES

We compiled phylogenies that represent composites of two recently published source phylogenies. Our primary (hereafter “main”) phylogeny includes a substantial sample of all coelurosaurian theropods (those more derived than Tyrannosauroidea) from compsognathids to maniraptorans, including Mesozoic Aves, reflecting the focus of the study. The phylogeny is complete for relatively well-represented fossil taxa, but it excludes those based on fragmentary fossils or whose validity is questioned. The main source phylogenies are a comprehensive tree of theropods (Turner *et al.* 2012), and a Mesozoic avian phylogeny (O'Connor and Zhou 2013). We further complemented the main composite tree by the addition of species missing from the source phylogenies, and by the resolution of polytomies. For the main phylogeny, we excluded *Jixiangornis* and *Shenzhouraptor* as they are considered possible synonyms of *Jeholornis* (Zhou and Li 2010). In our main tree, *Epidexipteryx* is classified as a nonavian paravian (Dececchi and Larrson 2013) and *Epidendrosaurus* is also excluded from the main analyses as it represents a juvenile (Zhang *et al.* 2002). Further, in the main analyses, *Microraptor zhouianus* is synonymous with *Microraptor gui* (Gong *et al.* 2012; Xing *et al.* 2013).

We tested the sensitivity of our analyses to alternative phylogenetic topologies and branch lengths with four additional trees: (1) the recent phylogeny of Godefroit et al. (2013), in which the new taxon *Aurornis* is regarded as the most basal bird, with a sister-group relationship between the Troodontidae and Aves; in this phylogeny no species are added, and any polytomies are reduced to descending species; (2) a revised version of the main tree with possible synonyms and juveniles added for completeness (“full”); (3) an alternative version of the main tree (called “arch”) with the single change of placing *Archaeopteryx* outside Aves to be the most basal member of the troodontids; and (iv) a final tree (“alt. branch”) based upon the full tree, and including *Eshanosaurus deguchiianus* (Barrett 2009), and used to test differences in branch lengths (discussed further below). Further details of the phylogenies are provided in the Supporting Information (Informal Supertree Construction) and the phylogenies are available at figshare (http://dx.doi.org/10.6084/m9.figshare.820135). The ages of all taxa in the study were collated by MJ Benton (MJB) from synoptic resources and current primary literature. In all trees, branch lengths were scaled to time using fossil occurrence dates of taxa in the tree (Brusatte *et al.* 2008), using a script written for R (http://www.graemetlloyd.com/methdpf.html). This method dates nodes according to their oldest descendant taxon, but this means that all nodes would contain zero branch lengths, even if the first occurrences in the fossil record are congruent with phylogeny, because each node is dated by its oldest descendant. Therefore, a unit of time is added to the descendant of the node from a preceding branch length to prevent zero-length branches (Brusatte *et al.* 2008). As the root length also contributes to the sharing of time between descendant branches, the root length can influence branch lengths elsewhere in the tree. For the main tree, the root branch length was set to either five or 10 million years, meaning that the root is placed at either 171 Ma or 176 Ma. These estimates are broadly in line with stratigraphy for the origin of coelurosaurians that are more derived than tyrannosauroids (Carrano *et al.* 2012). To test for the possibility that underestimated branch lengths might have an effect on rate calculations (e.g., leading to false inference of high rates), we added the dubious theropod *Eshanosaurus deguchiianus* from the Early Jurassic (Barrett 2009; Zanno 2010) to the main phylogeny, thereby placing the root of the tree back to around 200 Ma and having the effect of lengthening internal branches on the phylogeny. We consider the effects of *Eshanosaurus* to be extreme: the branch length leading to Paraves is almost 20× longer (6.08 Ma) for trees including, compared to those excluding, *Eshanosaurus* (0.31 Ma on the full tree).

As a further test of the influence of branch lengths on rate calculations, we undertook a sensitivity analysis using the timePaleoPhy function in the R package paleotree (Bapst 2013). In these analyses, we used the following settings: alternative branch-scaling methods, minimum-branch length (set to 2 Ma) in which branches are set to a minimum defined value and later branches are shortened to accommodate the true timing of diversification; additive branch length (with an additive value of 1 Ma), so 1 Ma is added to each branch; and equal, which is equivalent to the Graeme Lloyd script but here takes dates from a uniform distribution of the age range. For the main phylogeny, and the phylogeny based on Godefroit, we obtained a sample of five trees for each of these three branch-scaling methods; age ranges of taxa were taken from the Paleobiology database (Carrano *et al.* 2013).

### MORPHOLOGICAL CHARACTERS

Data were collected on the length of the femur and forelimb elements. Femur length is a widely used proxy for overall body size in theropods, which itself is a proxy for a large number of biologically relevant traits (Carrano 2006). Forelimb size is recorded as total humerus + forearm (radius or ulna) + manus lengths. All data were collected from published sources and the Paleobiology database (Carrano *et al.* 2013), and multiple measurements were recorded as means. Measurements for all elements were preferably taken from a single specimen, but as we wanted to maximize the number of taxa included, this was not always possible. Clearly there could be problems in assessing relative metrics if data for femur and forelimb lengths are taken from animals of very different body sizes. As a mitigation, the analyses were run twice, first with our compiled data (Carrano 2006, and other sources) and then with data from a single paper (Dececchi and Larrson 2013). The body size proxy from this source was snout-vent length (SVL), an alternative to femur length; although femur length is widely used as a proxy for body size in theropod dinosaurs (Carrano 2006), another view is that it may be a poor estimator of body size in Paraves, particularly among Aves (Dececchi and Larrson 2013). Prior to analysis, all data were log-transformed. All data and phylogenies are available at doi.org/10.6084/m9.figshare.820135.

We found morphological data on femur length from 125 species and on forelimb length from 76 species, of which 71 species had data on both femur and forelimb. Branch lengths were estimated on complete phylogenies, which were subsequently pruned to match the available data. The final trees with the effective tree size for femur (125 species), forelimb (76 species), and femur and forelimb (71 species) are shown (Fig. S1).

### MODELS OF TRAIT EVOLUTION

We modeled morphological evolution using four complementary phylogenetic comparative methods: (1) we estimated the phylogenetic position and magnitude of changes in the rate of evolution of body size and forelimb length using the trait MEDUSA method of Thomas and Freckleton (2012); (2) we compared the mode and rate of evolution for body size and forelimb among Paraves, Aves, and nonparavian Theropoda; (3) we assessed the fit of models of directional evolution of body size in Paraves and Aves respectively to test if miniaturization continued within either of these clades; and (4) we tested for changes in the coevolutionary relationship between body size and forelimb length among Paraves, Aves, and nonparavian Theropoda. Below we describe the models in detail.

#### i. Rates of evolution

The trait MEDUSA method tests for shifts in the rate of evolution on the branches of the phylogeny in which the location and magnitude of shifts are not known a priori (Thomas and Freckleton 2012). These shifts can either be clade-wide (shared by all branches of a clade), or on a single internal branch leading to the node that represents the most recent common ancestor of a clade. The trait MEDUSA algorithm starts with a baseline of a homogeneous Brownian motion model across the entire phylogeny, it then iterates across all nodes in the tree, allowing a different rate at each clade and individual branch in turn, to locate the shift that most improves the likelihood of the model. This single shift is fixed and is the starting point for the next step, where a second shift is located. This process continues up to a user-defined maximum number of shifts (Thomas and Freckleton 2012). The best overall model is assessed by comparison of the Akaike Information Criterion (AIC) among the best constant-rate, one-shift, two-shift and so on models. Alternative approaches to identifying shifts in evolutionary rates have been developed that use Reversible-Jump MCMC methods (Eastman *et al.* 2011; Venditti *et al.* 2011). The RJMCMC method of Eastman et al. (2011), as used by Benson and Choiniere (2013), only allows rate shifts to occur across whole clades. This is an important constraint, because shifts that occur along a single internal branch cannot be readily identified and the method may instead average such a high rate on a single branch across all descendant lineages, so leading to the false inference of high rates across whole clades. The RJMCMC method of Venditti et al. (2011) models both single-branch and clade shifts, but the current implementation is limited to univariate analyses. The trait MEDUSA model, as implemented in the R package MOTMOT can accommodate multivariate data. To do so, it models changes in covariance among traits, where all elements of the trait covariance matrix are modified by a single scalar such that the proportionality of the matrix is constant across the tree, that is, the eigenvectors (the correlations among traits) are constant but eigenvalues are proportional. Revell and Collar (2009) modeled multivariate evolution by allowing each element of the trait covariance matrix to vary among lineages, which increases the generality and potentially the biological realism of the model. However, their model requires that the locations of shifts are defined a priori and cannot be applied to shifts that occur along single internal branches in the phylogeny.

We used the function transformPhylo.ML with the tm2 algorithm in the R package MOTMOT (Thomas and Freckleton 2012) to fit the trait MEDUSA model. We fitted the model to (1) body size (univariate), (2) total forelimb length (univariate), and (3) body size + forelimb length (multivariate). We allowed up to five possible rate shifts (although in practice the best-fitting model always contained fewer shifts) and set a minimum clade size of five, which prevents the algorithm from searching for shifts in very small clades. To determine an appropriate ΔAICc threshold, a simulation was run in which BM was modeled on the phylogeny 1000 times, and the trait MEDUSA was then used to detect a single shift on these simulated data of BM. The 95th percentile of the difference between the AICc of the BM and single-shift model was then used as the AICc cut-off value (Thomas and Freckleton 2012). After simulation, this value was found to be 9.22; this means that during application of the tm2 algorithm, an additional rate shift that improves the AICc by <9.22 compared to a model with fewer shifts would be rejected.

An increase in evolutionary rate on a single branch constitutes either rapid change on that branch, or a parallel (directional) clade-wide shift in a trait value (Thomas and Freckleton 2012). As trait MEDUSA method cannot distinguish between these alternative evolutionary interpretations, below (ii and iii) we describe two complementary approaches to clarify these patterns.

#### ii. Ornstein–Uhlenbeck models

We used variants of the Ornstein–Uhlenbeck (OU) model of evolution (Hansen 1997; Butler and King 2004; Beaulieu *et al.* 2012) to test alternative evolutionary patterns among Paraves and Aves. We fitted alternative OU-based models using the R package OUwie (Beaulieu *et al.* 2012), which allows lineages to differ in three parameters: (1) θ, often referred to as the primary adaptive optimum; (2) α, variously referred to as the evolutionary pull toward those optima, or the strength of stabilizing selection; and (3) σ, the rate of stochastic evolution (Beaulieu *et al.* 2012). We fitted seven alternative evolutionary models to the femur and forelimb data respectively and compared model fit using AICc. We repeated the models allowing a shift in parameters among either Paraves or Aves. The models were as follows: (1) BM1, Brownian motion model, single σ, does not estimate α or θ; (2) BMS, two σs, does not estimate α or θ; (3) OU1, OU model, single θ, α, and σ; (4) OUM, two θs, single α and σ; (5) OUMV, two θs, two σs, single α; (6) OUMA, two θs, two αs, single σ; and (7) OUMVA, two θs, two σs, two αs. In all models, the ancestral state θ_0_ was not included, so in this model, the starting value of θ is estimated from an OU stationary distribution.

As we noted above, OU models are often presented in the context of stabilizing selection, but some of the parameters, particularly α, are difficult to interpret because statistical support for high α could arise from either evolution among lineages toward a shared optimum or from unaccounted error in the phylogeny or the data. We avoid speculative interpretation of α by treating it as statistical tool to account for deviation from Brownian motion, rather than a function of evolutionary process.

#### iii. Directional trends

High rates on single internal branches inferred using trait MEDUSA may be caused by high rates at the origin of that clade, or by directional changes of evolution across the clade away from the ancestral state. To test this possibility, we pruned the phylogenies to Paraves only, and compared different models of evolution within these clades. The null BM model was compared to a directional model of evolution where a significantly higher likelihood for a directional model indicates evolution away from the ancestral state of the clade (Pagel 1997, 1999). We used BayesTraits (Pagel and Meade 2013) and fitted a BM model (model A) and a directional (model B). We compared the fit of the models using likelihood ratio tests.

#### iv. Relationship between body size and forelimb length

Aves and Paraves are expected to show different relationships between body size and forelimb compared to nonparavian theropods. Specifically, Dececchi and Larrson (2013) infer that apparent forelimb elongation in Aves arose from an allometric move to a small body size. To test for variation in the relationship of femur and forelimb between major theropod groups, we used the phylogenetic generalized least squares (PGLS) function in the R package caper to perform an analysis of covariance (ANCOVA; Orme et al. 2011). This corrects for statistical nonindependence in trait values (Felsenstein 1985; Freckleton *et al.* 2002) by simultaneously estimating and correcting for the strength of phylogenetic signal in the model residuals using Pagel's λ (Pagel 1997, 1999). A λ value of 1 indicates strong phylogenetic signal (i.e., evolution of a trait is consistent with a constant-rate Brownian motion model), and a value of 0 indicates a lack of phylogenetic signal (deviation from Brownian motion). We fitted a series of alternative models, with interaction terms defined by major clades. Interaction terms were fitted to forelimb length and one of the discrete dummy variables to define either (i) Aves (0), other taxa (1); (ii) Paraves (0), other taxa (1); or (iii) Aves (0), other Paraves (1), other taxa (2). A statistically significant interaction term would imply that the slope of the relationship between femur and forelimb differs among major theropod taxa.

### SIMULATIONS

We used simulations to test for potential biases toward finding shifts in particular clades or branches. We note that this is not a test of the accuracy of the branch length reconstruction, but rather a test to determine whether any inferred rate shifts could be statistical artifacts. First, we simulated 1000 datasets under a constant-rate BM model on the main phylogeny pruned to 71 species, so as to match the number of species in the femur + forelimb dataset. Data simulated under these conditions are not expected to result in identification of major rate shifts. We then fitted the trait MEDUSA model to each simulated data vector and recorded the frequency and position of identified shifts. We paid particular attention to the short branch leading to Paraves, and recorded the node position of the identified shift as the number of nodes away from the Paraves where negative numbers indicate nodes outside Paraves and positive numbers indicate nodes within Paraves. If any bias toward identifying rate shifts on the Paraves branch exists, we expect the frequency of shifts to peak at node 0.

Second, we simulated data on the same tree with an increase in rates on the Paraves branch of 10, 50, 100, 500, or 1000 times the background rate. For each magnitude of rate shift, we simulated 1000 datasets and again fitted the trait MEDUSA model, recording the frequency, location, and magnitude of shifts.

## Results

### RATES OF CHARACTER EVOLUTION

#### Multivariate models of body size and forelimb length

**Figure 1:**
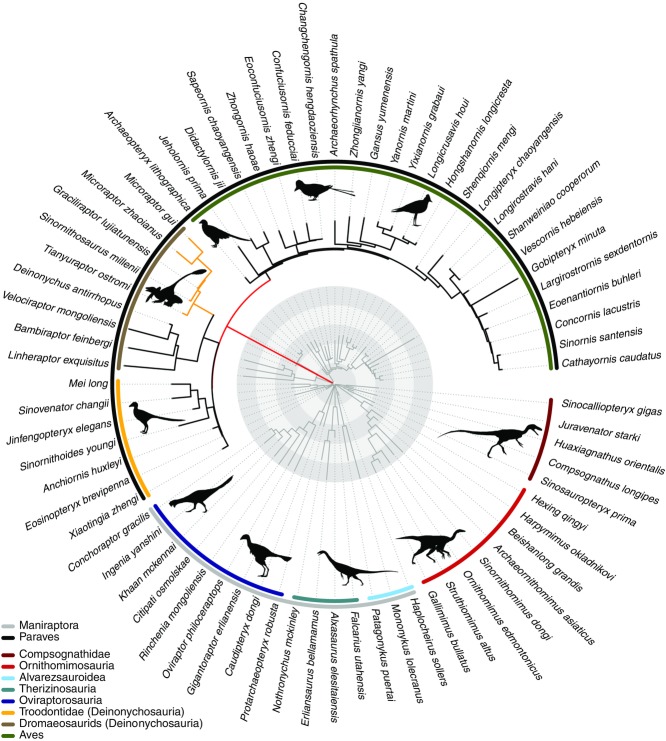
Rates of femur and forelimb evolution in Theropoda. Branch lengths are scaled, (i) red branch leading to the Paraves indicating an ∼200-fold rate increase relative to the background rate and (ii) yellow branches to Microraptorinae indicating an approximately eightfold rate increase relative to the background rate (not scaled relative to evolutionary rate). The original time-calibrated phylogeny is shown in dark gray. Circular rings indicate 5 Ma time intervals from the KPg boundary. Silhouettes drawn by Scott Hartman, Matt Martyniuk, Emily Willoughby, Jaime Headon, and Craig Dylke or modified by T. Michael Keesey were downloaded from http://phylopic.org.

The main phylogeny shows two increases in the multivariate data; a clade increase in Microraptorinae (8.52 × the background rate) and a larger branch-based increase leading to Paraves (166.4 × the background rate; AICc = −126.36, BM model AICc = −84.02; Fig. 1; Table 1). Indeed, the Paraves branch increase is recovered on most alternative phylogenies. One exception arises when using a phylogeny with extended branch lengths, and femur length as the body size proxy, although the increase is observed on this tree when using the SVL body size proxy (Table S5). Even on the “alt. branch” phylogeny showing no Paraves-branch increase at the AICc cutoff of 9.22, a shift is found in the Microraptorinae (7.60 × the background rate) at the ΔAICc of 9.22, and an increase on the Paraves branch is seen at a lower ΔAICc (1.8). Similarly, using the Dececchi and Larrson (2013) dataset, a clade increase in the dromaeosaurids (20.8 × the background rate) is observed (AICc = −48.28, BM AICc = 4.45), and a branch-based increase at the Paraves (40.92 × the background rate) is observed at a lower AICc cut-off (6.3, Table S5).

**Table 1:** Rates of continuous evolution in body size (a), forelimb length (b), and simultaneous analysis of body size and forelimb length (c) using trait MEDUSA

	Shift 1	Shift 2
(a) Body size (femur length)
Clade	Ornithomimidae	*Paraves*
Type	Clade	*Branch*
Rate	0.0310485306374238	*272.183712380408*
Lower CI	0.0107361619159907	*25.7279220940737*
Upper CI	0.146917741744914	*NA*
Model AICc	−8.630297	−*16.46549*
Single-rate AIC	2.903468	
(b) Forelimb length		
Clade	Enantiornithes	
Type	Clade	
Rate	0.202655937981583	
Lower CI	0.084093502295542	
Upper CI	0.573787236049022	
Model AICc	−13.8642	
Single-rate AIC	−7.775569	
(c) Simultaneous femur and forelimb length		
Clade	Microraptorines	Paraves
Type	Clade	Branch
Rate	8.5287339844581	166.401472957915
Lower CI	3.75495117178781	28.9116058496682
Upper CI	24.5239401626678	NA
Model AICc	−109.55345	−126.36988
Single-rate AIC	−84.02344	

The detected shifts for each analysis are shown in order of detection, along with the estimated rates, and a comparison of the multiple shift model AICc and the AICc for a single-rate model (Brownian Motion). The type refers to whether a shift is shared by all branches in a clades clade (“clade”) or a shift is found on a branch leading to that clade but is not shared by its descendants (“branch”). Results in italics show a result detected at the Paraves branch, but one that is not significant at the AICc cutoff.

AIC, Akaike information criterion.

#### Body size

The trait MEDUSA model identified a model with one distinct rate shift (AICc = −16.46) on the main phylogeny, a clade decrease in the Ornithomimidae (0.03 × the background rate). A branch-based increase leading to Paraves (272.2 × the background rate) is seen on most phylogenies, but it is not significant at the AICc cut-off of 9.22, and is seen at a lower cut-off value (7.7 on the main phylogeny). For full information on clade composition for all traits and phylogenies, see Tables S1–S5. On the Godefroit phylogeny, a clade decrease was found in the Troodontidae (0.024; AICc = 41.88, BM AICc = 50.8), and the second branch increase leading to the Paraves (118 × the background rate; AICc = 36.63). A branch-based increase on the branch leading to Paraves was detected as the second rate shift on most trees.

Similarly, an alternative body-size proxy, SVL, shows an increase on the branch leading to Paraves across most phylogenies (Table S4). On the main phylogeny, two increases were detected using the SVL data, a clade increase in dromaeosaurids (5.10 × the background rate), and the Paraves branch increase (592.7 × the background rate). This model had a superior AICc score (16.36) compared to the BM model (36.88, Table S4). On the full phylogeny (“alt.branch”) that incorporated information on *Eshanosaurus*, a branch-based increase leading to Paraves was not identified for femur data (Table S5). With the femur size proxy, a clade-based decrease is seen in Ornithimimidae on the “alt.branch” phylogeny (0.029). With the SVL body size proxy, a clade-based increase is seen in dromaeosaurids (4.91), but an increase was found at the Paraves branch (34.3 × the background rate), but only at a low ΔAICc threshold (0.6, Table S5).

#### Forelimb length

Across nearly all datasets and phylogenies, no increased or decreased rates (clade or branch-based) were found for forelimb length (Table 1). A general trend is for a clade decrease in the avian clade Enantiornithes, but this is not a consistent trend across all phylogenies and alternative branch lengths (Table S2), and is never found at the AICc cutoff of 9.22. On the main phylogeny, a rate decrease 0.20 × the background rate in Enantiornithes is detected (AICc = −13.8, BM model AICc = −7.7). A BM model is favored in the phylogeny with alternative branch lengths (Table S5).

#### Sensitivity analysis

As a large amount of change appears to occur at the origin of the Paraves, we tested how these observations changed with different branch-scaling methods in paleotree (Bapst 2013). These tests generally showed the change in body size + forelimb evolution is observed more consistently at the branch leading to the Paraves (Table S6) compared to the evolution of body size alone (Table S7). No increases are found for forelimb evolution alone (Table S8).

We also tested how long the Paraves branch would have to be to reject a rate shift for simultaneous analysis of body size and forelimb length. The Paraves branch was manually altered from 0 Ma to 500 Ma in length, and the likelihood of the BM model was recorded. At 1.92 log-likelihoods from the maximum likelihood (104 Ma) the branch length is 19 Ma; we interpret that the Paraves branch would have to be greater than 19 Ma in length for the shift on this branch to be nonsignificant.

### DIRECTIONAL EVOLUTION

As noted above, the trait MEDUSA algorithm cannot distinguish between rapid periods of evolution at the origin of a focal clade, and sustained periods of directional evolution among constituent lineages of the focal clade (Thomas and Freckleton 2012). Our results suggest that the detected rapid branch-based increases probably arise from rapid branch-specific evolution at the clade origin, as we find no evolution for directional trends in Paraves. On the main phylogeny, there is no significant difference in the likelihoods of the BM and Directional evolution models in Paraves (*P* = 0.23, df = 1, chi-square statistic = 1.41), and no significant trends were found on any of the phylogenies (Table S9).

### EVOLUTIONARY REGIMES IN PARAVES AND AVES

Paraves, rather than Aves alone, shifted to a different evolutionary model relative to other coelurosaurian theropods (Table 2). On all trees and for both femur and forelimb size, the model with a regime shift at Paraves, rather than Aves, is favored (Table S10). We found strong support for a reduction in femur length within Paraves (57.5 mm for Paraves, 147.9 mm for other coelurosaurians) with weaker evidence for a concurrent reduction in evolutionary rates (Table 2). Importantly, the best-fitting paravian regime-shift model for femur length is substantially better than the best-fitting avian regime shift model (ΔAICc = 6.15; Table 1). The paravian forelimb reduced slightly in size (Paraves = 158.4 mm; other taxa = 234.4 mm; Table 2). In contrast, the equivalent model that incorporates a reduction in rate within Aves (Table 2) is an inferior fit (ΔAICc = 2.48).

**Table 2:** Adaptive regimes for femur and forelimb evolution in (i) Paraves with the rest of the tree and (ii) Aves with the rest of the tree

	Model	AICc	AICc wt	α 1	α 2	σ 1	σ 2	θ 1	θ 2
Paraves femur	^*^OUMVA	−16.154	0.494	9.0 × 10^−16^	0.019	0.201	0.003	2.17 (0.08)	1.76 (0.08)
Aves femur	^*^OUMV	−10.068	0.529	0.021	-	0.004	0.002	2.04 (0.07)	1.64 (0.11)
Paraves forelimb	^*^OUMA	−10.875	0.497	9 × 10^−14^	0.024	0.002	-	2.37 (0.1)	2.20 (0.08)
Aves forelimb	^*^BMS	−8.398	0.272	-	-	0.003	0.001	2.26 (0.08)	-

Adaptive regimes are parameterized by the strength of pull (α) toward the state optima (θ), and the evolutionary rate (σ). The OUMVA models allow all parameters to differ, whereas all except α in the OUMV and σ in the OUMA can vary. The BM model (BMS) allows only σ to vary and does not estimate α.

AIC, Akaike information criterion.

For forelimb evolution, the results are the same across most phylogenies, with the best-fitting model being the OUMA model with a regime shift at Paraves (Table S10). However, the Godefroit phylogeny shows a different pattern, with no change in optimum between Paraves and non-Paraves (Table S10). Models with additional evolutionary regimes (split into Aves, Paraves, and other taxa) do not improve the fit of the two-split models (Table S11).

### RELATIONSHIP BETWEEN FEMUR AND FORELIMB LENGTH

**Figure 2:**
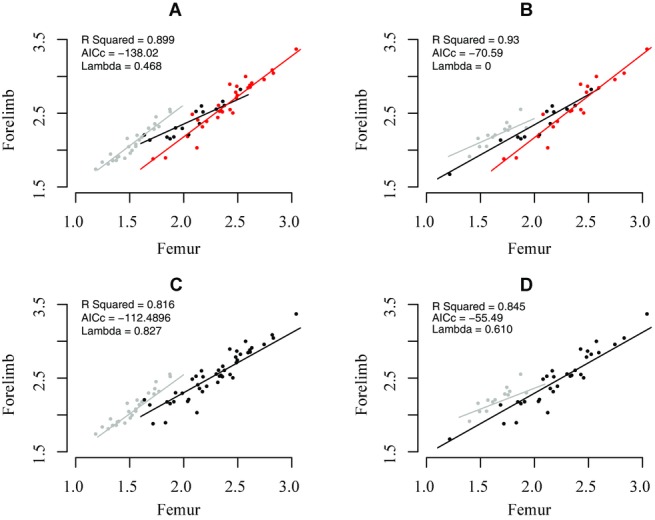
The relationship between body size and forelimb length. When the phylogeny is split into three regimes (A and B), Aves (gray), Paraves (black), and other theropods (red), differences are seen between the main phylogeny in the slope for Aves and the phylogeny of Godefroit et al. (2013) in which a shallower slope for Aves is seen. These differences appear to be due to species being moved from the Aves and Paraves; Godefroit et al. (2013) place *Xiaotingia* and *Anchiornis* in Paraves, whereas in the main phylogeny they are placed in Aves. When the phylogenies are split into two portions, Aves and non-Aves, (C and D) differences are once again seen between the trees. The main phylogeny, following conclusions from Dececchi and Larrson (2013), shows a smaller Aves intercept, indicative of an allometric change in scaling between body size and forelimb length (see Table 1). Again, differences are seen in the alternative phylogeny of Godefroit (D) where this lower Aves intercept is not found. Models that split the phylogenies into three portions (A and B) are better-fitting models (according to AICc scores) and explain more of the variance in the data (according to higher R-squared values) than models that split the phylogenies into Aves and non-Aves (C and D).

Evolutionary trajectories of femur–forelimb evolution differ among Aves, Paraves, and other coelurosaurians. The slope of the femur–forelimb relationship does not differ from unity within nonparavian coelurosaurians (Fig. 2; Table 3a). However, the slopes for Paraves and Aves depend on the tree and definition of taxa. Using the main phylogeny, at small body sizes, paravians have larger relative forelimb lengths than nonparavian coelurosaurs, whereas at large body sizes, paravians are predicted to have smaller relative forelimb lengths (Fig. 2). Within the observed data range, the majority of paravian taxa have a larger relative forelimb length than predicted from the nonparavian coelurosaurian regression line (Fig. 2A). However, when analyzed using the Godefroit et al. (2013) phylogeny, the relationship changes; in the model with three levels, Aves have a shallower slope and do not have a reduced intercept compared to the other groups (Fig. 2D; Table 3b).

**Table 3:** The relationship between body size and forelimb length

	Main phylogeny			Godefroit et al. (2013) phylogeny		
(a)	Coef. (±SE)	*t*	*P*	Coef. (±SE)	*t*	*P*
Int. Aves	0.396 (0.158)	2.50	<0.05	1.136 (0.257)	4.42	<0.001
Int. Paraves	0.627 (0.212)	2.95	<0.01	-0.426 (0.280)	−1.52	0.13
Int. Other	−0.411 (0.211)	−1.94	0.056	−1.227 (0.307)	−3.99	<0.001
Femur: Aves	1.108 (0.095)	11.66	<0.001	0.646 (0.150)	4.30	<0.001
Femur: Paraves	−0.443 (0.123)	−3.59	<0.001	0.170 (0.162)	1.05	0.299
Femur: other	−0.011 (0.113)	−0.098	0.921	0.484 (0.167)	2.89	<0.01
(b)						
Int. Aves	0.423 (0.209)	2.01	0.047	1.230 (0.304)	4.03	<0.001
Int. Other	0.251 (0.232)	1.08	0.283	0.566 (0.171)	3.30	<0.01
Femur: Aves	1.061 (0.120)	8.84	<0.001	−0.587 (0.319)	−1.83	0.073
Femur: other	−0.247 (0.132)	−1.87	0.064	0.259 (0.179)	1.44	0.155

PGLS ANOVA models for the relationship between femur and forelimb length including an interaction term with three levels designating major taxa—Aves, Paraves, and remaining taxa (other, a)—and an interaction term with two levels—Aves and remaining taxa (other, b). *P*-values show whether parameters differ from 0 (for Aves), or from Aves (Paraves and other). Also see Figure2.

PGLS, phylogenetic generalized least squares.

When the model is split into Aves and remaining taxa, Aves have a different relationship, with a lower intercept and steeper slope (Fig. 2C; Table 3b). Similarly, on the Godefroit et al. (2013) phylogeny, Aves have a higher intercept and a shallower slope (Fig. 2D; Table 3b). The model with three levels is a better-fitting model (AICc = −138.02) compared to alternative models with a split at Paraves alone (AICc = −127.06) and Aves alone (AICc = −112.48, Table S14).

The results may be affected by the differing phylogenetic positions of certain species that move between Paraves and Aves in different phylogenies (Fig. 2; Table 3). To ensure that differences between trees were not a result of different taxon sampling, the main phylogeny and Godefroit phylogeny were reduced to the same set of taxa (Table S12). In the reduced main tree, *Anchiornis* and *Xiaotingia* are classified as nonavian Paraves, but in the Godefroit tree they are classified as Aves. Changing classification of these taxa resulted in a shallower avian slope on the Godefroit phylogeny, and a steeper slope on the main phylogeny. However, these differences are not so pronounced when using the alternative dataset (Table S13).

### SIMULATIONS

As discussed above, the simulated data indicated an AICc cutoff of 9.22 was necessary for an acceptable 5% type 1 error rate. In addition to using simulations to indicate the appropriate AICc cutoff, we also used simulations to assess potential bias and power.

We find no evidence of statistical bias in the position of rate shifts; in particular, there was no evidence of falsely high rates of evolution on the short branch leading to Paraves (Fig. S2). Although the constant-rate model is rejected for 5% of simulated datasets for the phylogeny, the percentage of branch-based increases at the Paraves branch was 0.2%. Indeed, there was no detectable bias toward any specific node in either the larger or smaller tree.

When high rates of evolution are simulated on the branch leading to Paraves, they are generally accurately recorded, although the accuracy of the rate estimation is variable (Fig. S2). A branch rate of 500 times is needed for detection in just over 50% of trees and generally the estimation of rates is a slight underestimate.

## Discussion

Before the origin of Aves, on the branch leading to Paraves, high rates of evolution led to a smaller body size and a relatively larger forelimb in Paraves. These changes are on a single branch leading to Paraves, representing a shift to a new smaller size and larger forelimb at this point. Rapid miniaturization and relative forelimb elongation at the root of Paraves explain the similarities between early birds and other Paraves (Padian and Chiappe 1998; Turner *et al.* 2007; Clarke and Middleton 2008; Novas *et al.* 2012). As with previous studies, we find strong evidence for paravian miniaturization (Xu *et al.* 2003; Turner *et al.* 2007; Novas *et al.* 2012) and no trend for forelimb elongation (Dececchi and Larrson 2013), but the identification here of increased rates of evolution of size-dependent forelimb and body size at the origin of Paraves emerges from our novel analytical approach. As with a recent study (Deccechi and Larrson 2013), we find evidence for a different allometric relationship between forelimb size and body size in Aves, but this result is altered by different phylogenetic topologies, and we find little evidence for elevated rates leading to or within Aves.

### WHEN DID MORPHOLOGICAL CHANGES OCCUR?

We found that relative forelimb size evolves rapidly at the origin of Paraves, but that the relative elongation of the forelimb is primarily a consequence of dramatic reductions in body size, and when analyzed individually, there is no increased rate for forelimb evolution. We also present evidence that *Microraptor* underwent further modifications in the relationship between forelimb length and body size, although this is found with only one of the datasets; this may highlight, nonetheless, how experimentation with flight occurred in the Paraves (Dyke *et al.* 2013). Miniaturization in body size has been detected on this branch before (Turner *et al.* 2007; Novas *et al.* 2012), and it is generally known that paravians have larger forelimbs for their overall size than other theropods (Chatterjee and Templin 2004). Additionally, the lack of a trend for increasing forelimb evolution is widely recognized (Benson and Choiniere 2013; Dececchi and Larrson 2013). On the branch leading to the Paraves, a move to a smaller body size is consistent across phylogenies, but the power to detect a shift declines in cases with an extended branch leading to Paraves (Table S5) and on phylogenies constructed using alternative branch-scaling methods (Table S7). More consistent evidence is for the size-dependent shift in forelimb evolution at this point (Table S6). Elevated evolutionary rates on a branch leading to a clade, but not shared by descendants of that clade, have rarely been tested, but they may represent important macroevolutionary phenomena. For example, a recent study of mammalian body size evolution revealed high rates on branches leading to several clades (Venditti *et al.* 2011).

Testing for, and identification of, single-branch shifts can significantly change our understanding of macroevolutionary patterns. Here, we have identified a very rapid change in rate at the origin of Paraves. Previous studies using methods that can only detect clade-wide rate shifts, have inferred that the hindlimb evolved rapidly across much of Aves (Benson and Choiniere 2013). Although we note that Benson and Choiniere (2013) used PCA, other differences in their results are likely to relate to their methodology; the RJMCMC method (Eastman *et al.* 2011) used by Benson and Choiniere (2013) cannot detect branch-specific rate changes, whereas trait MEDUSA (Thomas and Freckleton 2012), used here, and an alternative RJMCMC method (Venditti *et al.* 2011) can.

Branch-based change implies either a quick change in the value of a trait or that the entire clade underwent shifts in the trait value in parallel (Thomas and Freckleton 2012); here we interpret this as a case of rapid evolution, as our subsequent analyses suggested no evidence for directional evolution or continued rapid change within Paraves (Table S10). Our OU models revealed reduced body size in Paraves coupled with low rates of evolution across the clade (Table 2). The reduction in body size is consistent with the high rate of evolution inferred by the trait MEDUSA analyses (Thomas and Freckleton 2012). Similarly, we found reductions in forelimb length among Paraves, but here not coupled with changes in rate (Table 2). Although our models support general trends toward smaller body size, this does not preclude evolution toward large size within a clade; indeed, some paravians did reach large sizes (Turner *et al.* 2007), and there is an increased rate of body size evolution for dromaeosaurids (Table 1).

### RELATIONSHIP BETWEEN FORELIMB LENGTH AND BODY SIZE

Changes in scaling relationship between the theropods, Paraves, and Aves (Fig. 2A) suggest a different scaling relationship in the Paraves and Aves compared to the remaining theropods. Therefore, our results are in line with a different avian scaling relationship between forelimb and body size (Dececchi and Larrson 2013), but also further differences in the Paraves; changes in the Paraves are also reflected in changes in the rate of evolution. If Aves have a different relationship between forelimb length and body size, an expectation may be for further changes in rates of evolution leading to Aves. However, on only one phylogeny with one specific dataset is there an increased rate for body size dependent evolution of forelimb length (Table S3), so there is scant evidence for higher rates leading to Aves. One reason for this may be that the forelimb ratios of early Aves are very similar to those of their close relatives (Middleton and Gatesy 2000), or that changes occurred over an extended time. However, methods that have analyzed discrete character evolution have found high evolutionary rates at the base of Aves and Paraves (Lloyd *et al.* 2012; S. Brusatte, pers. comm.).

Our best-fitting models (based on AICc scores) indicate differences in slope of the femur–forelimb relationship between three groups (Aves, Paraves, and nonparavian theropods); Aves have a shallower slope than the two other groups. However, these differences in slope are contingent on the placement of two taxa, *Xiaotingia* and *Anchiornis*. These are normally classed as Deinoncyhosauria (Lee and Worthy 2012; Turner *et al.* 2012), whereas Godefroit et al. (2013) class them within Aves. Analysis of the main tree, in which *Xiaotingia* and *Anchiornis* are placed in Deinonychosauria, shows that the Paraves have a shallower slope than nonparavians, suggesting that the allometric relationship between forelimb and femur shifted sharply at the origin of Paraves. This appears to have been followed by a subsequent shift back to a nonparavian allometry within Aves. In contrast, based on the Godefroit et al. (2013) phylogeny, we find that the allometric slope becomes progressively shallower toward Aves (Fig. 2; Table 3). These differences between phylogenies do not appear to be dependent on dataset or size (Tables S12 and S13), but instead indicate that the phylogenetic placement of a few taxa can profoundly influence our understanding of the coevolutionary trajectories of traits related to flight. We note, with an alternative dataset, that the patterns are similar, although with less pronounced differences.

### METHODOLOGICAL APPROACHES

Studies of morphological rates of evolution have focused mainly on single characters, but measurements of multiple traits may be important and may reflect a more accurate picture of evolution, especially in relation to body size dependent evolution. Here, we measured rates of evolution in two characters simultaneously to study changes in covariance between trait values; by testing changes in covariance it is possible to build a more detailed picture of the relationships between traits in a single analysis rather than comparing rates in isolation. Alternative methods include the use of residuals from regressions (Revell 2009), Principal Components Analysis (Benson and Choiniere 2013), or ratios. One limitation of the trait MEDUSA method is that changes in rate are assumed to apply proportionally to the trait covariance matrix, whereas traits might well evolve at different rates in such a way that the trait covariance matrix could change disproportionately. The change in body size (as measured by femur or SVL length) is not matched by any change in the rate of forelimb evolution (Table 1); this suggests a change in covariance at this point. Furthermore, this result is more consistently found (Table S6), rather than body size evolution alone (Table S7).

Methods that use BM as a model of trait evolution, as here, assume evolution occurs as a gradual process. BM (random walk) is commonly used as a null model in comparative phylogenetics (Freckleton *et al.* 2002; O'Meara *et al.* 2006; Thomas *et al.* 2009; Venditti *et al.* 2011), but it is increasingly recognized that evolutionary rates (tempo) are underpinned by the evolutionary model (mode) that is used to inform them; models that use an incorrect mode of evolution can incorrectly judge rates of evolution (Hunt 2012; Slater 2013). An issue here could be that the high branch-based rates leading to Paraves might have been caused by periods of directional selection that led to the perceived high rates (Gingerich 2001, 2009; Hunt 2012), rather than strictly elevated rates. However, tests of directional evolution among the Paraves suggest that this explanation is unlikely. Further, BM rates are sampled from a normal distribution, so high or low rates may be difficult to detect (Landis *et al.* 2013). The trait MEDUSA method used here is one of a suite of models that incorporates rate changes by allowing branch lengths to vary to accommodate rate variation under BM (see also, Eastman *et al.* 2011; Venditti *et al.* 2011; Revell *et al.* 2012; Thomas and Freckleton 2012; Landis *et al.* 2013). However, it has been suggested that modeling changes in the mode, rather than rate, may be more effective in understanding models of evolution (Slater 2013). A corollary to this suggestion is that localized changes in the rate of evolution can result in models of evolutionary mode being misled; for example, a small number of rate shifts toward the tips of the tree can result in falsely favoring an OU model. Although caution must be taken with the assumptions of evolutionary models (Hunt 2012; Slater 2013), a wide range of evidence suggests that evolutionary rates were indeed higher leading to Paraves.

Improvements could be made to the current analyses and methods. Although we attempted to accommodate alternative topologies and include appropriate species, we recognize that coelurosaurian phylogeny is in a state of flux. Further, as a proxy for body size, bone lengths in extinct taxa are widely used (Sookias *et al.* 2012), but a problem may be that lengths do not represent true body size (Campione and Evans 2012), and in particular changes in posture in Aves mean that use of the femur can be problematic (Dececchi and Larsson 2013). However, our findings were validated by use of an alternative measure for body size (SVL), but alternatives such as bone circumferences are also available (Campbell and Marcus 1992).

## Conclusions

The origin of Aves and the origin of flight have been widely discussed (e.g., Padian and Chiappe 1998; Allen *et al.* 2013; Benson and Choiniere 2013; Dececchi and Larrson 2013). Key patterns are supported here: miniaturization of the Paraves (Turner *et al.* 2007; Novas *et al.* 2012), no change in forelimb length evolution, and a shift in the allometry between body size and forelimb length (Dececchi and Larrson 2013). Main changes happen at the origin of the Paraves; there is little evidence for heightened rates of evolution within or leading to Aves, although birds do appear to have a different body size and forelimb relationship. Overall, morphological changes associated with flight, reduction in body size and elongation of the forelimb, occurred before the origin of Aves. Simple morphological changes will not be able to explain the origins of flight (Hutchinson and Allen 2009), but these changes support the notion that changes related to flight represent adaptations. Although previous discussions have focused on whether flight evolved from the trees down (e.g., Chatterjee and Templin 2004; Xu *et al.* 2003, 2011) or from the ground up (Padian and Chiappe 1998), the close association of different flight adaptations among paravians may support the notion of paravian-wide experiments in gliding flight (Dial 2003; Dyke *et al.* 2013).

## DATA ARCHIVING

The doi for our data is doi.org/10.6084/m9.figshare.820135.

## Supporting Information

Figure S1 Phylogenetic trees used for analysis. **Figure S2**. Simulations validate the performance of the trait MEDUSA (tm2 algorithm) method. **Table S1**. Trait MEDUSA (tm2 algorithm) for femur length (body size) across all phylogenies. **Table S2**. Trait MEDUSA (tm2 algorithm) for rates of forelimb length evolution across all phylogenies. **Table S3**. Trait MEDUSA (tm2 algorithm) for rates of body size (femur length) and forelimb length evolution (measured simultaneously) across all phylogenies. **Table S4**. Trait MEDUSA (tm2 algorithm) for rates of body size, forelimb length, and simultaneous body size and forelimb length evolution using alternative data (Dececchi and Larrson 2013). **Table S5**. Trait MEDUSA (tm2 algorithm) for rates of body size, forelimb length, and simultaneous body size and forelimb length evolution using a phylogeny with alternative branch lengths to the main phylogeny. **Table S6**. Results from trait MEDUSA of simultaneous femur and forelimb evolution using different branch-scaling methods from the R packages paleotree. **Table S7**. Results from the trait MEDUSA measure of body size evolution using different branch-scaling methods from the R packages paleotree. **Table S8**. Results from the trait Medusa measure of forelimb evolution using different branch-scaling methods from the R packages paleotree. **Table S9**. Tests of directional evolution performed on pruned phylogenies representing Paraves and Aves extracted from different phylogenies. **Table S10**. Results from the OUwie models (BM1, BMS, OU1, OUM, OUMV, OUMA, OUMVA) across phylogenies indicating changes in the optimum between the “theropods” on the tree, and either the Aves or the Paraves. **Table S11**. A model of a split in the Aves, Paraves, and other for various OUwie models (BMS, OU1, OUM, OUMV, OUMA, OUMVA) for body size (femur length) and forelimb length). **Table S12**. PGLS models to clarify that differences between the main phylogeny and Godefroit et al. (2013) phylogeny are not due to sample size (a), and a lot of the differences can be explained by reclassification of just two taxa (*Anchiornis* and *Xiaotingia*) when phylogenies are reduced to the same size (b). **Table S13**. Use of alternative data sources for body size and forelimb length (Dececchi and Larrson 2013). **Table S14**. A PGLS model of 3 regimes (Aves, other Paraves, and other taxa) with interactions between femur and forelimb length is the best-fitting model.
