# Supplementary material for: HIGH RATES OF EVOLUTION PRECEDED THE ORIGIN OF BIRDS
Source: Evolution. 2014 Feb 23;68(5):1497–510. doi: 10.1111/evo.12363 (PMC4289940; doi:10.1111/evo.12363)
Supplement: Figure S1 — Phylogenetic trees used for analysis. Figure S2. Simulations validate the performance of the trait MEDUSA (tm2 algorithm) method. Table S1. Trait MEDUSA (tm2 algorithm) for femur length (body size) across all phylogenies. Table S2. Trait MEDUSA (tm2 algorithm) for rates of forelimb length evolution across all phylogenies. Table S3. Trait MEDUSA (tm2 algorithm) for rates of body size (femur length) and forelimb length evolution (measured simultaneously) across all phylogenies. Table S4. Trait MEDUSA (tm2 algorithm) for rates of body size, forelimb length, and simultaneous body size and forelimb length evolution using alternative data (Dececchi and Larrson 2013). Table S5. Trait MEDUSA (tm2 algorithm) for rates of body size, forelimb length, and simultaneous body size and forelimb length evolution using a phylogeny with alternative branch lengths to the main phylogeny. Table S6. Results from trait MEDUSA of simultaneous femur and forelimb evolution using different branch-scaling methods from the R packages paleotree. Table S7. Results from the trait MEDUSA measure of body size evolution using different branch-scaling methods from the R packages paleotree. Table S8. Results from the trait Medusa measure of forelimb evolution using different branch-scaling methods from the R packages paleotree. Table S9. Tests of directional evolution performed on pruned phylogenies representing Paraves and Aves extracted from different phylogenies. Table S10. Results from the OUwie models (BM1, BMS, OU1, OUM, OUMV, OUMA, OUMVA) across phylogenies indicating changes in the optimum between the “theropods” on the tree, and either the Aves or the Paraves. Table S11. A model of a split in the Aves, Paraves, and other for various OUwie models (BMS, OU1, OUM, OUMV, OUMA, OUMVA) for body size (femur length) and forelimb length). Table S12. PGLS models to clarify that differences between the main phylogeny and Godefroit et al. (2013) phylogeny are not due to sample size (a), and a lot of the di [file evo0068-1497-SD1.docx]

**Supplementary Material**

**Table of contents**

Phlylogenies..................................................................................................................................2-7

Informal supertree construction............................2-4

Main phylogenies.................................................5-7

Supplementary Results...............................................................................................................8-28

Trait Medusa models..........................................8-19

Ornstein Uhlenbeck models..............................20-25

PGLS models....................................................26-28

Supplementary References............................................................................................................29

**Phylogenies**

**Informal Supertree Construction**

As discussed in the main text the main phylogenies used for analyses are formed using an informal supertree approach by combining the phylogeny of Turner et al. (2012) and O’Connor and Zhou (2013) further species were then added to the phylogeny. Here, we provide additional details on how additional taxa were added to the phylogeny.

*Compsognathidae*

Species present in Turner et al. (2012) have been resolved further according to Senter et al. (2007). We follow the revised cladogram for *Juravenator starki* (Butler and Upchurch 2007) in placing this taxon at the base of Maniraptora, close to *Scipionyx samniticus* and *Sinocalliopteryx gigas*, rather than as a compsognathid.

*Ornithomimidae*

As in Turner et al. (2012), but *Hexing qingyi* and *Beishanlong grandi* added according to (Liyong et al. 2012). Resolved according to (Liyong et al. 2012), with *H. qingyi* and *Shenzhousaurus orientalis*, and *B. grandis* and *Harpymimus okladnikovi* assumed to be sister-species. *Ornithomimus velox* and *O. edmontonicus* added according to (Lloyd et al. 2008).

*Alvarezsauridae*

*Bonapartenykus ultimus* added according Agnolin et al. (2012). Remaining species resolved using Agnolin et al. (2012), although without *Ceratonykus* and *Xixianykus* included. *Xinjiangovenator parvus* and *Bagaraatan ostromi* – as in (Lloyd et al. 2012), as a basal deriving clade to the Therizinosauria (although this placement of *Bagaraatan* is problematic, S. Brusatte pers. comm.).

*Therizinosauria*

*Falcarius utahensis, Beipiaosaurus inexpexctus, Martharaptor greenriverensis, Alxasaurus elesitaiensis, Nanshiungosaurus brevispinus, Suzhousaurus megatherioides, Nothronychus mckinley* and *Segnosaurus galbinensis* all added and resolved according to Senter et al. (2012a). *Neimongosaurus yangi* placed as more derived than *Erliansaurus bellamamus* as in Senter (2007) to resolve the polytomy in Senter et al. (2012a). *Enigmosaurus mongoliensis* placed as basal to the *Suzhousaurus* as in Zanno et al. (2009).

*Oviraptoridae*

*Guyedong maniraptoran* placed in close affinity to *Protarchaeoptryx robusta* (Lloyd et al. 2008), as with *E. deguchiianus* it is both included and excluded from analsysis. *Caudipteryx dongi , Caudipteryx zoui* added as sister species (Carrano 2006), *Caenagnathus collinsi, Avimimus portentosus, Elmisaurus rarus, Hagryphus giganteus, Chirostenotes pergracilis, Gigantoraptor erlianensis, Oviraptor philoceraptops, Rinchenia mongoliensis, Citipati osmolskae, Khaan mckennai, Conchoraptor gracilis, Nemegtomaia barsboldi, Machairasaurus leptonychus, Heyuannia huangi, Ingenia yanshini* all added according to Longrich et al. (2010). *N. barsboldi* and *M. leptonychus* assumed to be descending taxa. *Nomingia* and *Microvenator celer* added as in Lloyd et al. (2008). *E. rarus* and *H. giganteus* assumed to be sister-species next to *C. pergracilis* (Senter 2007).

*Aves*

*Jixiangornis* *orientalis* and *Shenzhoraptor* *sinesis* may be synonyms of *Jeholornis prima*, but some consider them to be distinct (O’Connor and Zhou 2013). Although *J. prima* and *J. orientalis* may be synonymous (Wang et al. 2013), we maintain them as distinct, but closely-related species. *Confuciusornis feducciai* added as sister group to *Confuciusornis sanctus* (Zhang et al. 2009), and *Changchenornis hengdaoziensis* considered to be an outgroup to the remaining clades. *Protopteryx feningensis* resolved as the most basal enantiornithine (Zhang and Zhou 2000), and *Jeibinia luanhera* added as a basal enantiornithine basal to *Shanweinio cooperum*. *Gobiopteryx minuta* and *Pengornis houi* are neighbouring taxa (Turner et al. 2012), so the polytomy between *S. cooperum* and *Longirostravis hani* is assumed to be a sister-group. *Vescornis hebeiensis* is then assumed to be basal to *G. minuta* (Wang and Zhang 2011). *Bohainornis guoi* added as sister to *Eoenantiornis walkeri* as it is the most similar taxon (Hu et al. 2009). *Cathayornis caudatus* and *Sinornis santensis* assumed to be sister taxa (MJB). *Iaceornis marshi* as in Clarke et al. (2006), and Longrich (2009). *Limenavis patagonica* and *Enaliornis baretti* assumed to be sister-species.

*Scansioripterygidae*

In the full tree, *Epidendrosaurus* (Zhang et al. 2002) is included, but is excluded from the other trees for being a juvenile (Zhang et al. 2002).

*Deinonychosauria*

*Eosinopteryx breivpenna* (Godefroit et al. 2013b) is included in the trees as a basal troodontid. *Geminiraptor suarezarum* is placed in basal position next to *Sinovenator changii* (Senter et al. 2010). *Sinusonasus* *magnodens* placed as more derived than *Sinornithoides youngi* (Xu and Wang 2004). *Dornogov troodontid* as in Lloyd et al. (2008). *Talos sampsoni* placed as basal to *Sinorthoides youngi* (one of three equally parsimonious explanations; Zanno et al. 2011). *Linhevenator* placed as closest to *Troodon* *formosus* (Xu et al. 2011).

*Unenlagia* *comahuensis* and *Unenlagia* *paynemilli* placed in sister-species relationship. *Neuquenraptor argentinus* is described as basal (Novas and Pol 2005), and Lloyd et al. (2008) has it as the most basal of the microraptorine-type clade, so it is placed there. *Hesperonychus elizabethae*, *Gracilliraptor lujiatunensis*, *Microraptor* spp., *Sinornithosaurus milleni*, and *Tianyuraptor ostromi* resolved according to Senter et al. (2012b), and done fully by assuming *Cryptovolans pauli* is sister to the *Microraptor* clade (as they are sometimes considered to be synonymous; Burnham et al. 2011), so *S. milleni* and *G lujiatunensis* are sister-species (Senter et al. 2012). *Microraptor hanqingi* added according to Gong et al. (2012) and *M*. *gui* from Xu et al. (2003).

*Adasaurus mongoliensis* added according to Senter et al. (2012b). *Achillobator giganticus*, *Utahraptor ostromaysorum*, and *Dromaeosaurus albertensis* resolved as in Senter (2007). The position of *Yurgovichia doellingi* is unknown exactly (Senter er al. 2012b), therefore it is added as a basal member of the dromaeosaurinae clade. *Linheraptor exquistus* is placed as intermediate between basal and derived dromaeosaurids near to *Tsaagan mangas*, as stated in Xu et al. (2010) *Sinorthinolestes langstoni* and *Velociraptor* spp. (*V. mongoliensis* and *V. osmolskae*) are assumed to be sister taxa. *Belaur bondoc* is closest to *Velociraptor* (Csiki et al. 2010) and *Deinonychus antirrhopus* being more basal to these members, and *A. mongoliensis* being the most basal member (Senter 2007).

**Main Phylogenies**

**Figure S1. Phylogenetic trees used for analysis. The final trees used in the analyses represented the main tree for species with femur data (a), forelimb data (b) and femur and forelimb data (c).**

**(a)**


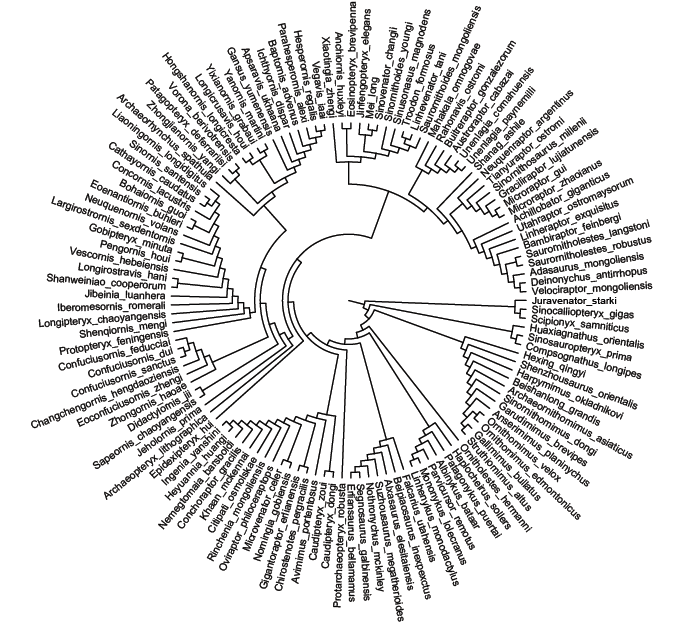


**(b)**

**
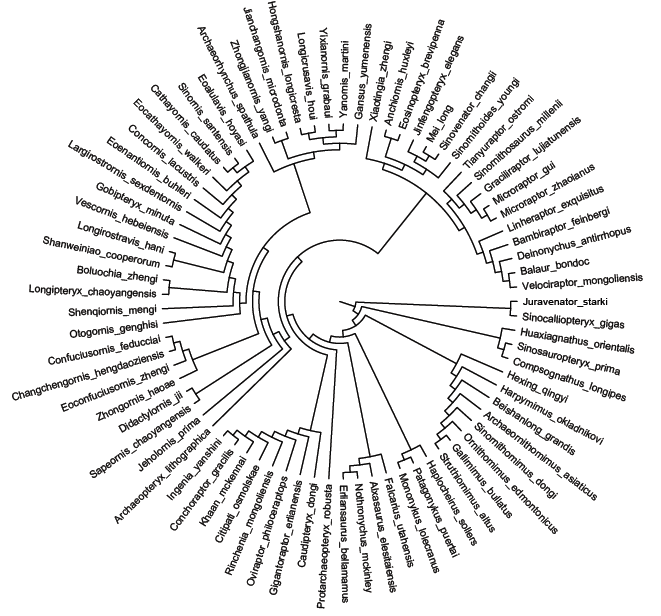
**

**(c)**

**
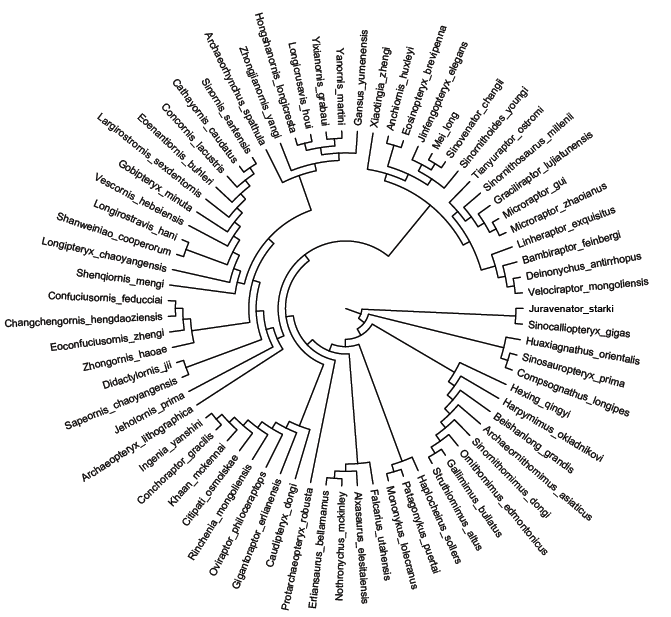
 Supplementary Results**

**Rates of character evolution**

In all tables ‘clade’ refers to a rate shift being shared across all branches of a clade, and ‘branch’ refers to a shift in the branch leading to the node of the most recent common ancestor of a clade.

**Table S1.** Trait MEDUSA (tm2 algorithm) for femur length (body size) across all phylogenies. The phylogenies are named in capitals, and the order in which shifts occur are in shown in order from right to left. Type refers to whether a shift is a clade based shift, or a branch-based shift leading to a clade. The final AICc of the model is given along with the AICc of the single rate (Brownian Motion) model. Italicised text shows the next inferred shift in rate but where the shift failed to meet the AICc cut-off value 9.22.

|  | Shift 1 | Shift 2 |
| --- | --- | --- |
| MAIN | **Ornithomimidae** | ***Paraves*** |
| Type | Clade | *Branch* |
| Rate | **0.0310485306374238** | ***272.183712380408*** |
| Lower CI | 0.0107361619159907 | *25.7279220940737* |
| Upper CI | 0.146917741744914 | *NA* |
| Model AICc | -8.630297 | *-16.46549* |
| Single-Rate AICc | 2.903468 |  |
|  |  |  |
| FULL | **Ornithomimidae** | ***Paraves*** |
| Type | Clade | *Branch* |
| Rate | **0.0267342606747853** | ***280.01728020179*** |
| Lower CI | 0.0092507436062325 | *27.0246809609066* |
| Upper CI | 0.126639490479054 | *NA* |
| Model AICc | 4.936508 | *-3.431078* |
| Single-Rate AICc | 17.228732 | |
|  |  | |
|  |  |  |
| ARCH | **Ornithomimidae** | ***Paraves*** |
| Type | Clade | *Branch* |
| Rate | **0.0263002864399732** | ***320.947466091029*** |
| Lower CI | 0.00909990067769905 | *31.0422238320635* |
| Upper CI | 0.124602977964685 | *NA* |
| Model AICc | 9.893256 | *1.349277* |
| Single-Rate AICc | 22.275546 | |
|  |  |  |
| MAIN (ROOT 10) | **Ornithomimidae** | ***Paraves*** |
| Type | Clade | *Branch* |
| Rate | **0.0293065895199591** | ***128.325628031057*** |
| Lower CI | 0.0101315430056203 | *8.67941960761174* |
| Upper CI | 0.138774200696244 | *NA* |
| Model AICc | -13.926898 | *-16.932274* |
| Single-Rate AICc | -2.739482 | |
|  |  |  |
| GODEFROIT | **Troodontidae** | ***Paraves*** |
| Type | Clade | *Branch* |
| Rate | **0.0244234284764583** | ***118.683781206064*** |
| Lower CI | 0.00733912013154531 | *10.9977231967774* |
| Upper CI | 0.162925478248095 | *NA* |
| Model AICc | 41.88189 | *36.63301* |
| Single-Rate AICc | 50.83301 | |

**Table S2.** Trait MEDUSA (tm2 algorithm) for rates of forelimb length evolution across all phylogenies. The phylogenies are named in capitals, and the order in which shifts occur are in shown in order from right to left. Type refers to whether a shift is a clade based shift, or a branch-based shift leading to a clade. The final AICc of the model is given along with the AICc of the single rate (Brownian Motion) model. Italicised text shows the next inferred shift in rate but where the shift failed to meet the AICc cut-off value 9.22.

|  | Shift 1 |
| --- | --- |
| MAIN | ***Enantiornithes*** |
| Type | *Clade* |
| Rate | ***0.202655937981583*** |
| Lower CI | *0.084093502295542* |
| Upper CI | *0.573787236049022* |
| Model AICc | *-13.8642* |
| Single-Rate AICc | -7.775569 |
|  |  |
| FULL | ***Enantiornithes*** |
| Type | *Clade* |
| Rate | ***0.164738802357272*** |
| Lower CI | *0.0694818349144498* |
| Upper CI | *0.464337815735289* |
| Model AICc | *-6.505464* |
| Single-Rate AICc | 1.547666 |
|  |  |
| ARCH | ***Enantiornithes*** |
| Type | *Clade* |
| Rate | ***0.154757231947609*** |
| Lower CI | *0.0668845486627756* |
| Upper CI | *0.431374962104038* |
| Model AICc | *-6.199038* |
| Single-Rate AICc | 2.534501 |
|  |  |
| MAIN (ROOT 10) | ***Microraptorines*** |
| Type | *Clade* |
| Rate | ***4.38521330669294*** |
| Lower CI | *1.47101266917724* |
| Upper CI | *21.4021756233112* |
| Model AICc | *-14.60583* |
| Single-Rate AICc | -8.902668 |
|  |  |
| GODEFROIT | ***Troodontidae + Aves*** |
| Type | *Clade* |
| Rate | ***0.261168827274184*** |
| Lower CI | *0.115620359354654* |
| Upper CI | *0.619877221152777* |
| Model AICc | *17.73392* |
| Single-Rate AICc | 24.15138 |

**Table S3.** Trait MEDUSA (tm2 algorithm) for rates of body size (femur length) and forelimb length evolution (measured simultaneously) across all phylogenies. The phylogenies are named in capitals, and the order in which shifts occur are in shown in order from right to left. Type refers to whether a shift is a clade based shift, or a branch-based shift leading to a clade. The final AICc of the model is given along with the AICc of the single rate (Brownian motion).

|  | Shift 1 | Shift 2 |
| --- | --- | --- |
| MAIN | **Microraptorines** | **Paraves** |
| Type | Clade | Branch |
| Rate | **8.5287339844581** | **166.401472957915** |
| Lower CI | 3.75495117178781 | 28.9116058496682 |
| Upper CI | 24.5239401626678 | NA |
| Model AICc | -109.55345 | -126.36988 |
| Single-Rate AICc | -84.02344 |  |
|  |  |  |
| FULL | **Microraptorines** | **Paraves** |
| Type | Clade | Branch |
| Rate | **7.48737424964631** | **375.217323201451** |
| Lower CI | 3.49433277797773 | 67.1189412366813 |
| Upper CI | 19.4629004315712 | NA |
| Model AICc | -110.93759 | -131.70881 |
| Single-Rate AIC | -86.18092 |  |
|  |  |  |
| ARCH | **Microraptorines** | **Paraves** |
| Type | Clade | Branch |
| Rate | **6.698473180176** | **383.272004030195** |
| Lower CI | 3.13290261149882 | 66.8942698323141 |
| Upper CI | 17.3896559828484 | NA |
| Model AICc | -98.68154 | -117.19067 |
| Single-Rate AIC | -76.49896 |  |
|  |  | |
| MAIN (ROOT 10) | **Microraptorines** | **Paraves** |
| Type | Clade | Branch |
| Rate | **8.75811501963516** | **80.3641114580877** |
| Lower CI | 3.85729351986804 | 12.3326507285088 |
| Upper CI | 25.1786908682723 | NA |
| Model AICc | -117.94015 | -127.28590 |
| Single-Rate AIC | -88.85803 |  |
|  |  |  |
| GODEFROIT | **Paraves** | **Aves** |
| Type | Branch | Branch |
| Rate | **168.856415638996** | **64.713985577921** |
| Lower CI | 28.2230877997797 | 10.5120307266786 |
| Upper CI | NA | NA |
| Model AICc | -22.60619 | -33.26341 |
| Single-Rate AIC | -13.42270 |  |

**Table S4.** Trait MEDUSA (tm2 algorithm) for rates of body size, forelimb length, and simultaneous body size and forelimb length evolution using alternative data (Dececchi and Larrson 2013). SVL (snout-ventral length) refers to the alternative body size proxy, and FRL (forelimb) to alternative forelimb length. The phylogenies are named in capitals, and the order in which shifts occur are in shown in order from right to left. The final AICc of the model is given along with the AICc of the single rate (Brownian Motion). Italicised text shows the next inferred shift in rate but where the shift failed to meet the AICc cut-off value 9.22.

|  | Shift 1 | Shift 2 |
| --- | --- | --- |
| MAIN SVL | **Dromaeosaurids** | **Paraves** |
| Type | Clade | Branch |
| Rate | **5.10183847538044** | **592.717153587909** |
| Lower CI | 2.36902343719973 | 66.6857740413587 |
| Upper CI | 12.4062431599732 | NA |
| Model AICc | 30.34118 | 16.36031 |
| Single-Rate AIC | 36.88014 |  |
|  |  |  |
| MAIN FRL | NA |  |
| Type | NA |  |
| Rate | NA |  |
| Lower CI | NA |  |
| Upper CI | NA |  |
| Model AICc | 4.319553 |  |
| Single-Rate AIC | 4.319553 |  |
|  |  |  |
| MAIN SVL-FRL | **Dromaeosaurids 2** | **Paraves** |
| Type | Clade | Branch |
| Rate | **18.7849364000523** | **788.313917288862** |
| Lower CI | 8.35871403576726 | 149.518573177912 |
| Upper CI | 47.8600127239747 | NA |
| Model AICc | -31.600055 | -60.096613 |
| Single-Rate AIC | -3.193172 |  |
|  |  |  |
| GODEFROIT SVL | ***Paraves*** |  |
| Type | *Branch* |  |
| Rate | ***202.810216875485*** |  |
| Lower CI | *17.3798568880829* |  |
| Upper CI | *NA* |  |
| Model AICc | *17.11944* |  |
| Single-Rate AIC | 24.96002 |  |
|  |  |  |
| GODEFROIT FRL | ***Troodontidae + Aves (Godefroit)*** | |
| Type | *Clade* |  |
| Rate | ***0.135252898729689*** |  |
| Lower CI | *0.04777030310008* |  |
| Upper CI | *0.47201657754899* |  |
| Model AICc | *5.324505* |  |
| Single-Rate AIC | 11.45620 |  |
|  |  |  |
| GODEFROIT SVL-FRL | **Paraves** |  |
| Type | Branch |  |
| Rate | **263.591144794512** |  |
| Lower CI | 43.3815127070023 |  |
| Upper CI | NA |  |
| Model AICc | -42.56225 |  |
| Single-Rate AIC | -25.60089 |  |

**Table S5.** Trait MEDUSA (tm2 algorithm) for rates of body size, forelimb length, and simultaneous body size and forelimb length evolution using a phylogeny with alternative branch lengths to the main phylogeny. The phylogeny is the tree that includes *Eshanosaurus* (named Alt.Branch in the figure) and thus extending the branch leading to the Paraves (described in methods in the main text). Results are shown for this phylogeny using both the original data (named femur and forelimb in the figure) and the alternative dataset (named SVL and FRL in the figure). Similarly, for the alternative measure of body size (snout-ventral length) an increase is seen at a lower AICc cut-off as the next shift. The phylogenies are named in capitals, and the order in which shifts occur are in shown in order from right to left. Type refers to whether a shift is a clade based shift, or a branch-based shift leading to a clade. The final AICc of the model is given along with the AICc of the single rate (Brownian Motion) model. Italicised text shows the next inferred shift in rate but where the shift failed to meet the AICc cut-off value 9.22.

|  | Shift 1 | Shift 2 |
| --- | --- | --- |
| ALT. BRANCH FEMUR | **Ornithomimidae** |  |
| Type | Clade |  |
| Rate | **0.0295423140283263** |  |
| Lower CI | 0.0102255825692052 |  |
| Upper CI | 0.139816088612761 |  |
| Model AICc | -6.495243 |  |
| Single-Rate AIC | 4.649546 |  |
|  |  |  |
| ALT. BRANCH FORELIMB | NA |  |
| Type | NA |  |
| Rate | NA |  |
| Lower CI | NA |  |
| Upper CI | NA |  |
| Model AICc | -4.635224 |  |
| Single-Rate AIC | -4.635224 |  |
|  |  |  |
| ALT. BRANCH FEMUR-FORELIMB | **Microraptorinae** | ***Paraves*** |
| Type | Clade | *Branch* |
| Rate | **7.38834861637529** | ***22.8446455017845*** |
| Lower CI | 3.45842604687125 | *2.44517785310718* |
| Upper CI | 19.1713366313381 | *NA* |
| Model AICc | -124.10941 | *-125.91991* |
| Single-Rate AIC | -89.97507 |  |
|  |  |  |
| ALT. BRANCH SVL | **Dromaeosaurids** | ***Paraves*** |
| Type | Clade | *Branch* |
| Rate | **4.91897412392122** | ***34.3652385003896*** |
| Lower CI | 2.28882269522279 | *2.16995841845297* |
| Upper CI | 11.9474455661401 | *NA* |
| Model AICc | 21.0385 | *20.40347* |
| Single-Rate AIC | 36.98366 | *36.98366* |
|  |  |  |
| ALT. BRANCH FRL | **NA** |  |
| Type | NA |  |
| Rate | **NA** |  |
| Lower CI | NA |  |
| Upper CI | NA |  |
| Model AICc | 10.58856 |  |
| Single-Rate AIC | 10.58856 |  |
|  |  |  |
| ALT. BRANCH SVL-FRL | **Dromaeosaurids 2** | ***Paraves*** |
| Type | Clade | *Branch* |
| Rate | **20.8143570681501** | ***40.9269776797875*** |
| Lower CI | 9.37681628279471 | *6.07738983233015* |
| Upper CI | 52.6621539322078 | *NA* |
| Model AICc | -54.5479 | *-48.289360* |
| Single-Rate AIC | 4.453287 |  |

**(b) Definitions of taxon names**

**Paraves**- Scansoriopterygidae, Aves and Deinonychosauria

**Ornithomimidae** – *Garudimimus brevipes, Anserimimus planinychus, Ornithomimus velox, Ornithomimus edmontonicus, Gallimimus bullatus, Struthiomimus altus*

**Dromaeosaurids –** *Mahakla omnogovae ­– Velociraptor mongoliensis*

**Dromaeosaurids 2** – *Tianyuraptor ostromi, Sinornithosaurus millenii, Microraptor gui, Microraptor zhaoianus, Linheraptor exquisitus, Bambiraptor feinbergi, Deinonychus antirrhopus*

**Microraptorines** - *Tianyuraptor ostromi, Sinornithosaurus millenii, Graciliraptor lujiatunensis, Microraptor gui, Microraptor zhaoianus*

**Enantiornithes** – *Longipteryx chaoyangensis, Boluochia zhengi ,Shanweiniao cooperorum, Longirostravis hani, Vescornis hebeiensis, Gobipteryx minuta ,*

*Largirostrornis sexdentornis, Eoenantiornis buhleri, Concornis lacustris, Eocathayornis walkeri, Eoalulavis hoyasi, Cathayornis caudatus, Sinornis santensis*

**Troodontidae** - *Anchiornis huxleyi, Eosinopteryx brevipenna, Sinornithoides youngi, Jinfengopteryx_elegans, Mei_long, Sinovenator changii*

**Troodontidae (Godefroit)** – *Sinovenator, Sinusonasus, Sinornithoides, Sauornithoides, Troodon*

**Troodontidae + Aves (Godefroit)** – *Jinfengopteryx*-*Yixiangornis/Gansus*

**Aves (Godefroit) –** *Xiaotingia* – *Gansus*

**Table S6.** Results from trait MEDUSA of simultaneous femur and forelimb evolution using different branch-scaling methods from the R packages paleotree. Branches were scaled with paleotree using (i) the minimum branch length model (mbl) set to 2 million years, (ii) the all branches additive model (aba) with 1 Ma added to all branches, and (iii) the equal model with the root divergence increased by 5 million years. A sample of five phylogenies was made for each method for the ‘main’ and ‘Godefroit’ phylogenies, and rates are tested with the two datasets. The number of times a branch increase leading to the Paraves is shown, as well as any other significant shifts.

| Method | Data | Phlyogeny | Paraves Branch | All Significant Shifts |
| --- | --- | --- | --- | --- |
| MBL  2 MA | Main Data | Main Phylogeny | None significant. Paraves shift still first detected shift with a mean rate of 45.6. | None |
| ABA  1 Ma | Main Data | Main Phylogeny | 4/5 Paraves shifts significant.  Mean rate of 64.17 | 4/5 Paraves shifts |
| Equal  5 Ma | Main Data | Main Phylogeny | 5/5 Paraves shifts significant Mean rate of 214.12 | 5/5 Paraves increases |
| MBL  2 MA | Dececchi & Larrson data (SVL & FRL) | Main Phylogeny | 3/5 Paraves shifts.  Mean rate of 108.6.  First shift on all phylogenies | 3/5 Paraves shifts |
| ABA  1 MA | Dececchi & Larrson data (SVL & FRL) | Main Phylogeny | 5/5 Paraves shifts significant Mean of 146.85 | 5/5 Paraves shifts  1/5 Dromaeosaur branch increase (4.4x rate) |
| Equal  5 Ma | Dececchi & Larrson data (SVL & FRL) | Main Phylogeny | 5/5 Paraves shifts significant  Mean rate 541.75  First shift on all phylogenies. | 5/5 Paraves shifts |
| MBL  2 MA | Main Data | Godefroit Phylogeny | None significant.  Paraves shift second-detected shift on 4/5 phylogenies. | None |
| ABA  1 MA | Main Data | Godefroit Phylogeny | 3/5 Paraves significant  Mean rate of 90.93 | 3/5 Paraves shifts |
| Equal  5 Ma | Main Data | Main Phylogeny | 5/5Paraves shifts significant  Mean rate 202.56 | 5/5 Paraves shifts |
| MBL  2 MA | Dececchi & Larrson data (SVL & FRL) | Godefroit Phylogeny | No significant shifts, but Paraves first shift on all trees with a mean rate of 87.84. | None |
| ABA  1 MA | Dececchi & Larrson data (SVL & FRL) | Godefroit Phylogeny | 5/5 Paraves shifts significant  Mean rate of 141.23 | 5/5 Paraves shifts |
| Equal  5 Ma | Dececchi & Larrson data (SVL & FRL) | Godefroit Phylogeny | 5/5 Paraves shifts significant Mean rate of 350.44 | 5/5 Paraves shifts |

**Table S7.** Results from the trait MEDUSA measure of body size evolution using different branch-scaling methods from the R packages paleotree. Branches were scaled with paleotree using (i) the minimum branch length model (mbl) set to 2 million years, (ii) the all branches additive model (aba) with 1 Ma added to all branches, and (iii) the equal model with the root divergence increased by 5 million years. The ‘main’ and ‘Godefroit’ phylogeny branches are scaled using these methods, and rates are tested with the two datasets. The number of times a branch increase leading to the Paraves is shown, and any other significant shifts.

| Branch Method | Phylogeny | Data | Paraves Shift | Significant Shifts |
| --- | --- | --- | --- | --- |
| MBL 2 Ma | Main | Main Data | No | 5/5 Ornithomimidae clade decrease. Mean rate of 0.01 |
| ABA 1 Ma | Main | Main Data | No | 5/5 Ornithomimidae clade decrease. Mean rate of 0.01 |
| Equal 5 Ma | Main | Main Data | 3/5 Paraves shifts significant  Mean rate of 297  Second shift on all phylogenies | 5/5 Ornithomimidae clade decreases. Mean rate of 0.01  3/5 Paraves shifts |
| MBL 2 Ma | Main | Dececchi & Larrson Data | Second shift on 3/5 phylogenies (not significant) | No Significant Shifts |
| ABA 1 MA | Main | Dececchi & Larrson | Paraves increase detected on all phylogenies | No Significant Shifts |
| Equal 5 Ma | Main | Dececchi & Larrson Data | 5/5 Paraves shifts significant.  Mean rate of 391.6 | Paraves (5/5) |
| MBL 2 Ma | Godefroit | Main Data | No | One significant shift a slowdown in the Aves and Troodontids (clade) |
| ABA 1 MA | Godefroit | Main Data | No | 1/5 A slowdown in the in the Paraves (clade decrease)  Rate = 0.30. |
| Equal 5 Ma | Godefroit | Main Data | 2/5 Paraves shifts significant  Mean rate of 160.59  Shift seen on 4/5 | 2/5 Paraves Shifts |
| MBL 2 Ma | Godefroit | SVL | No | None |
| ABA 1 MA | Godefroit | SVL | No | None |
| Equal 5 MA | Godefroit | SVL | 1/5 Paraves shifts significant (196.9)  Paraves first detected shift on each phylogeny | 1/5 Paraves shift |

**Table S8**. Results from the trait Medusa measure of forelimb evolution using different branch-scaling methods from the R packages paleotree. Branches were scaled with paleotree using (i) the minimum branch length model (mbl) set to 2 million years, (ii) the all branches additive model (aba) with 1 Ma added to all branches, and (iii) the equal model with the root divergence increased by 5 million years. The ‘main’ and ‘Godefroit’ phylogeny branches are scaled using these methods, and rates are tested with the two datasets. The number of times a branch increase leading to the Paraves is shown, and any other significant shifts.

| Branch Method | Phylogeny | Data | Paraves Shift | Significant Shifts |
| --- | --- | --- | --- | --- |
| MBL 2 Ma | Main | Main Data | No | 4/5 Slowdown in Enantiornithes (clade).  Mean Rate = 0.1 |
| ABA 1 Ma | Main | Main Data | No | 2/5 Slowdown in Enantiornithes (clade)  Mean Rate = 0.11 |
| Equal 5 Ma | Main | Main Data | No | None |
| MBL 2 Ma | Main | Dececchi & Larrson Data | No | None |
| ABA 1 MA | Main | Dececchi & Larrson | No | None |
| Equal 5 Ma | Main | Dececchi & Larrson Data | No | None |
| MBL 2 Ma | Godefroit | Main Data | No | None |
| ABA 1 MA | Godefroit | Main Data | No | None |
| Equal 5 Ma | Godefroit | Main Data | No | None |
| MBL 2 Ma | Godefroit | Dececchi & Larrson Data | No | None |
| ABA 1 MA | Godefroit | Dececchi & Larrson | No | None |
| Equal 5 MA | Godefroit | Dececchi & Larrson Data | No | None |

**Directional evolution**

**Table S9.** Tests of directional evolution performed on pruned phylogenies representing Paraves and Aves extracted from different phylogenies. The p values reported are from the log-likelihood ratio test comparing the likelihoods of the Directional model (model B) and the null Brownian Motion model (model A) in BayesTraits.

| P value | | Likelihood BM | Likelihood Directional | Beta Trait 1 |
| --- | --- | --- | --- | --- |
| Main Tree | |  |  |  |
| Paraves | 0.2341518 | 10.805871 | 11.513602 | 0.001872326947 |
| Aves | 0.254283 | 16.827904 | 17.477713 | 0.002361944923 |
| Main Tree (root 10) | |  |  |  |
| Paraves | 0.2870253 | 10.792186 | 11.358947 | 0.001690502809 |
| Aves | 0.254283 | 16.827904 | 17.477713 | 0.002361944923 |
| Full Tree | |  |  |  |
| Paraves | 0.2793671 | 4.762518 | 5.347601 | 0.00189233009 |
| Aves | 0.252336 | 18.019501 | 18.674663 | 0.002328345079 |
| Arch | |  |  |  |
| Paraves | 0.2401591 | 2.481449 | 3.171279 | 0.002055811947 |
| Aves | 0.1963606 | 16.624614 | 17.459233 | 0.002794108098 |
| Godefroit | |  |  |  |
| Paraves | 0.4702161 | -8.144519 | -7.883745 | 0.001683187952 |
| Aves | 0.9532297 | -0.338828 | -0.337108 | 0.000210746126 |
| Main Tree SVL | |  |  |  |
| Paraves | 0.6245776 | -8.364321 | -8.244578 | 0.00148357871 |
| Aves | 0.3318071 | 2.865642 | 3.336558 | -0.00461284993 |
| Alternative Branch Phylogeny | |  |  |  |
| Paraves | 0.520268 | 8.452576 | 8.865938 | 0.001567648298 |
| Aves | 0.4338252 | 18.269981 | 18.882542 | 0.001619494962 |

**Figure S2.** Simulations validate the performance of the trait MEDUSA (tm2 algorithm) method. Simulated and fitted models using the full theropod phylogeny reduced to only species with femur/forelimb data (71). Data were simulated according to either Brownian motion (a) or an increase in the rate of evolution in the branch leading to the Paraves at 10x (b), 50x (c), 100x (d), 500x (e), and 1000x (f) the background rate. The mean rate of all shifts, the proportion of times a Paraves shift was found (i.e was the highest ranking single shift, regardless of ΔAICc) , and significant Paraves shifts (where a shift was detected with the ΔAICc cut-off of 9.22) are shown after searching for one shift in trait Medusa.

**
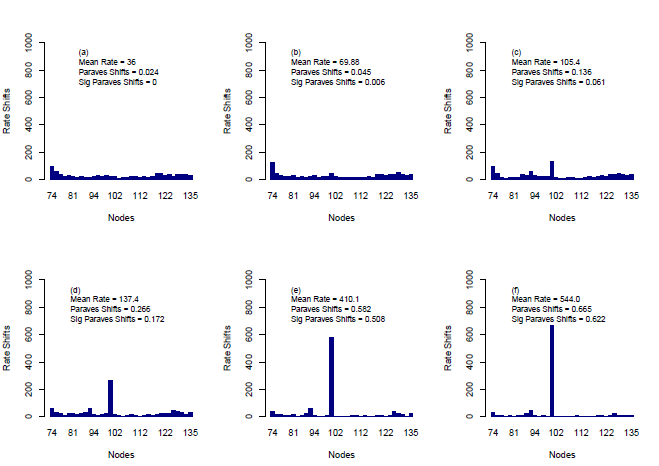
**

**Ornstein-Uhlenbeck models**

**Table S10.** Results from the OUwie models (BM1, BMS, OU1, OUM, OUMV, OUMA, OUMVA) across phylogenies indicating changes in the optimum between the ‘theropods’ on the tree, and either the Aves or the Paraves. The BM1 and BMS models fit a Brownian motion model to the phylogeny and allow for either a constant BM model in the tree (BM1) or different Brownian rates (sigma) in different regimes (BMS). Similarly the single rate OU models allow for a single OU model throughout the tree (OU1) or different OU selective optima in the different regimes. The OUMV allows for different stochastic rates and optima, the OUMA model allows for different optima and selective evolution (alpha), in the OUMVA model there are different adaptive rates, optima, and stochastic rates. With the estimates of optima for different clades the confidence intervals are also shown in brackets. The phylogenies on which analyses were performed are ‘main’, ‘full’, ‘arch’, and Godefroit.

|  | **Model** | **AICc** | **AICc wt** | **α 1** | **α 2** | **σ 1** | **σ 2** | **θ 1** | **θ 2** |
| --- | --- | --- | --- | --- | --- | --- | --- | --- | --- |
| **Main Paraves Femur** | **BM1** | 2.899546 | 3.6 9e-5 | - | - | 0.00262 | - | 1.96 [0.07] | - |
|  | **BMS** | 4.999453 | 1.2 8e-5 | - | - | 0.00262 | 0.00262 | 1.92 [0.07] | - |
|  | **OU1** | -8.649085 | 0.01160 | 0.02040 | - | 0.003 | - | 2.02 [0.06] | - |
|  | **OUM** | -7.185829 | 0.00558 | 0.01788 | - | 0.00366 | - | 2.08 [0.07] | 1.83 [0.1] |
|  | **OUMV** | -5.244002 | 0.00211 | 0.01871 | - | 0.00409 | 0.00350 | 2.07 [0.07] | 1.84 [0.09] |
|  | **OUMA** | -16.11984 | *0.48610* | 9.0e-16 | 0.01676 | 0.25763 | 0.00257 | 2.17 [0.09] | 1.75 [0.08] |
|  | ****OUMVA*** | *-16.15431* | 0.49455 | *9.0e-16* | *0.01992* | *0.20198* | *0.00306* | *2.17 [0.08]* | *1.76 [0.08]* |
| **Main Aves Femur** | **BM1** | 2.899546 | 0.00080 | - | - | 0.00262 | - | 1.96 [0.07] | - |
|  | **BMS** | 0.0136376 | 0.00342 | - | - | 0.00311 | 0.00167 | 1.96 [0.08] | - |
|  | **OU1** | -8.649085 | 0.26028 | 0.02040 | - | 0.00389 | - | 2.02 [0.06] | - |
|  | **OUM** | -4.910519 | 0.04014 | 0.01878 | - | 0.00381 | - | 2.01 [0.06] | 1.64 [0.14] |
|  | ****OUMV*** | *-10.06898* | *0.52939* | *0.02119* | *-* | *0.00490* | *0.00228* | *2.04 [0.07]* | *1.64 [0.11]* |
|  | **OUMA** | -3.212542 | 0.01717 | 0.0178 | 0.0278 | 0.00374 | 0.00374 | 2.03 [0.06] | 1.65 [0.12] |
|  | **OUMVA** | -7.530271 | 0.14876 | 0.0254 | 0.0254 | 0.00543 | 0.00543 | 2.06 [0.07] | 1.64 [0.10] |
| **Main Paraves Forelimb** | **BM1** | -7.782006 | 0.10595 | - | - | 0.00253 | 0.00253 | 2.26 [0.07] | - |
|  | **BMS** | -5.760129 | 0.03855 | - | - | 0.00273 | - | 2.26 [0.08] | - |
|  | **OU1** | -8.076625 | 0.12276 | 0.01018 | - | 0.00317 | - | 2.30 [0.07] | - |
|  | **OUM** | -5.829902 | 0.03992 | 0.01015 | - | 0.00317 | - | 2.31 [0.08] | 2.26 [0.12] |
|  | **OUMV** | -5.462697 | 0.03322 | 0.01645 | - | 0.00510 | 0.00286 | 2.31 [0.09] | 2.29 [0.11] |
|  | ****OUMA*** | *-10.87514* | *0.49747* | *9e-14* | *0.024413* | *0.00269* | *0.00269* | *2.37 [0.1]* | *2.20 [0.08]* |
|  | **OUMVA** | -8.632531 | 0.16210 | 9e-14 | 0.025925 | 0.00252 | 0.00284 | 2.37 [0.09] | 2.20 [0.08] |
| **Main Aves Forelimb** | **BM1** | -7.782006 | 0.20055 | - | - | 0.00253 | 0.00253 | 2.26 [0.07] | - |
|  | ****BMS*** | *-8.398187* | *0.27291* | *-* | *-* | *0.00305* | *0.00174* | *2.26 [0.08]* | *-* |
|  | **OU1** | -8.076625 | 0.23238 | 0.01018 | - | 0.00317 | - | 2.30 [0.07] | - |
|  | **OUM** | -5.663989 | 0.06955 | 0.00914 | - | 0.00310 | - | 2.30 [0.07] | 2.25 [0.19] |
|  | **OUMV** | -7.16807 | 0.14753 | 0.01199 | - | 0.00413 | 0.00211 | 2.31 [0.08] | 2.24 [0.16] |
|  | **OUMA** | -3.384277 | 0.02224 | 0.00891 | 0.01109 | 0.00309 | 0.00309 | 2.30 [0.07] | 2.25 [0.18] |
|  | **OUMVA** | -5.18794 | 0.05481 | 0.00002 | 0.00002 | 0.00423 | 0.00208 | 2.31 [0.08] | 11.8 [10.5] |
| **Full Tree**  **Paraves**  **femur** | **BM1** | 17.36721 | 6.58e-6 | - | - | 0.00305 | - | 1.94790 (0.08) | - |
|  | **BMS** | 19.03292 | 2.86e-6 | - | - | 0.00270 | 0.00328 | 1.95983 (0.07) | - |
|  | **OU1** | 2.330065 | 0.0121 | 0.02377 | - | 0.00470 | - | 2.02038 (0.06) | - |
|  | **OUM** | 3.242043 | 0.0076 | 0.02038 | - | 0.00434 | - | 2.08279 (0.07) | 1.80469 (0.10) |
|  | **OUMV** | 5.406402 | 0.0026 | 0.02027 | - | 0.00430 | 0.00430 | 2.08323 (0.07) | 1.80359 (0.10) |
|  | **OUMA** | -3.065431 | 0.1801 | 1.0e-14 | 0.01786 | 0.00300 | - | 2.17405 (0.09) | 1.71966 (0.09) |
|  | ****OUMVA*** | *-6.040031* | 0.7973 | *1.0e-14* | *0.02277* | *2.0e-03* | *0.0038* | *2.17405 (0.08)* | *1.7394 (0.08)* |
| **Full Tree**  **Aves**  **femur** | **BM1** | 17.36721 | 2.09e-05 | - | - | 0.00305 | - | 1.94790 (0.08) | - |
|  | **BMS** | 9.902133 | 0.0008 | - | - | 0.00381 | 0.00381 | 1.94577 (0.09) | - |
|  | **OU1** | 2.330065 | 0.0385 | 0.02377 | - | 0.00470 | - | 2.02038 (0.06) | - |
|  | **OUM** | 6.403008 | 0.0050 | 0.02235 | - | 0.00464 | - | 2.03287 (0.07) | 1.65796 (0.14) |
|  | ****OUMV*** | *-3.499423* | *0.7108* | *0.02505* | *-* | *0.00624* | *0.00238* | *2.04288 (0.08)* | *1.65395 (0.10)* |
|  | **OUMA** | 8.348324 | 0.0019 | 0.02132 | 0.02876 | 0.00458 | - | 2.02934 (0.07) | 1.66411 (0.13) |
|  | **OUMVA** | *-1.351123* | 0.2428 | *0.02461* | *0.02828* | 0.00618 | 0.00235 | 2.04168 (0.08) | 1.65669 (0.10) |
| **Full Tree**  **Paraves**  **forelimb** | **BM1** | 1.541448 | 0.0092 | - | - | 0.00301 | - | 2.22226 (0.08) | - |
|  | **BMS** | 3.693781 | 0.0031 | - | - | 0.00296 | 0.00304 | 2.22331 (0.08) | - |
|  | **OU1** | 0.5060406 | 0.0155 | 0.01196 | - | 0.00388 | - | 2.27021 (0.08) | - |
|  | **OUM** | 1.464909 | 0.0096 | 0.01167 | - | 0.00379 | - | 2.32203 (0.09) | 2.12774 (0.12) |
|  | **OUMV** | 3.365795 | 0.0037 | 0.01543 | - | 0.00490 | 0.00370 | 2.32083 (0.09) | 2.15687 (0.11) |
|  | ****OUMA*** | *-2.552831* | 0.*9223* | *9.0e-14* | *0.02441* | *0.00269* | *-* | *2.37986 (0.10)* | *2.20113 (0.09)* |
|  | **OUMVA** | -1.187196 | 0.0362 | *9.0e-14* | *0.02493* | *0.00253* | *0.00359* | 2.37980 (0.10) | 2.11204 (0.09) |
| **Full Tree**  **Aves**  **forelimb** | **BM1** | 1.541448 | 0.0583 | - | - | 0.00301 | - | 2.22226 (0.08) | - |
|  | **BMS** | -0.356107 | 0.1506 | - | - | 0.00419 | 0.00183 | 2.21049 (0.09) | - |
|  | **OU1** | 0.5060406 | 0.0978 | 0.01196 | - | 0.00388 | - | 2.27021 (0.08) | - |
|  | **OUM** | 2.690481 | 0.0328 | 0.01141 | - | 0.00382 | - | 2.27904 (0.08) | 2.16051  (0.16) |
|  | ****OUMV*** | *-2.418054* | 0.4223 | *0.02613* | *-* | *0.00827* | *0.00256* | *2.33174 (0.01)* | *2.22506 (0.01)* |
|  | **OUMA** | 4.945843 | 0.1193 | 0.01101 | 0.01208 | 0.00379 | - | 2.27776 (0.08) | 2.16117 (0.15) |
|  | **OUMVA** | 0.1210913 | 0.1186 | 0.03820 | 0.02934 | 0.01118 | 0.00337 | 2.39255 (0.09) | 2.21268 (0.10) |
| **Arch**  **Paraves**  **femur** | **Model** | **AICc** | **ΔAICc** | **α 1** | **α 2** | **σ 1** | **σ 2** | **θ 1** | **θ 2** |
|  | **BM1** | 8.412703 | 2.03e-05 | - | - | 0.00269 | - | 1.96568 (0.07) | - |
|  | **BMS** | 10.50754 | 7.13e-06 | - | - | 0.00266 | 0.00271 | 1.96692 (0.07) | - |
|  | **OU1** | -3.211926 | 0.0067 | 0.01899 | - | 0.00391 | - | 2.01000 (0.06) | - |
|  | **OUM** | -2.875199 | 0.0057 | 0.01809 | - | 0.00378 | - | 2.08026 (0.07) | 1.82627 (0.09) |
|  | **OUMV** | -0.8893887 | 0.0021 | 0.01927 | - | 0.00422 | 0.00367 | 2.07372 (0.07) | 1.83631 (0.09) |
|  | **OUMA** | -11.53665 | 0.4364 | 1.00e-15 | 0.01691 | 0.00265 | - | 2.17509 (0.09) | 1.74801(0.08) |
|  | ****OUMVA*** | *-11.99495* | *0.5488* | *3.00e-15* | *0.02027* | *0.00202* | *0.00319* | *2.17526 (0.08)* | *1.75958 (0.07)* |
| **Arch**  **Aves**  **femur** | **BM1** | 8.412703 | 0.0001 | - | - | 0.00269 | - | 1.96568 (0.07) | - |
|  | **BMS** | 4.742085 | 0.0010 | - | - | 0.00322 | 0.00163 | 1.96101 (0.08) | - |
|  | **OU1** | -3.211926 | 0.0578 | 0.01899 | - | 0.00391 | - | 2.01000 (0.06) | - |
|  | **OUM** | -2.110483 | 0.0333 | 0.02066 | - | 0.00402 | - | 2.04318 (0.06) | 1.65681 (0.12) |
|  | ****OUMV*** | *-8.156828* | *0.6855* | *0.02316* | *-* | *0.00523* | *0.00231* | *2.05189 (0.07)* | *1.65346 (0.09)* |
|  | **OUMA** | -2.076118 | 0.0327 | 0.01773 | 0.04303 | 0.00391 | - | 2.03302 (0.07) | 1.66187 (0.07) |
|  | **OUMVA** | -5.582191 | 0.1892 | 0.02759 | 0.02781 | 0.00577 | 0.00250 | 2.06755 (0.07) | 1.64953(0.08) |
| **Arch**  **Paraves**  **forelimb** | **BM1** | -7.211246 | 0.0608 | - | - | 0.00250 | - | 2.26927  (0.07) | - |
|  | **BMS** | -5.303549 | 0.0234 | - | - | 0.00276 | 0.00230 | 2.26439 (0.08) | - |
|  | **OU1** | -8.054088 | 0.0926 | 0.01104 | - | 0.00320 | - | 2.30751 (0.07) | - |
|  | **OUM** | -5.848893 | 0.0307 | 0.01119 | - | 0.00321 | - | 2.32484 (0.08) | 2.26123(0.11) |
|  | **OUMV** | -6.265022 | 0.0378 | 0.01903 | - | 0.00570 | 0.00284 | 2.32674 (0.09) | 2.29039 (0.09) |
|  | ****OUMA*** | *-11.68516* | *0.5694* | *1.00e-15* | *0.02669* | *0.00269* | *-* | *2.38115 (0.10)* | *2.21556 (0.08)* |
|  | **OUMVA** | -9.435616 | 0.1849 | 1.00e-15 | 0.02813 | 0.00252 | 0.00283 | 2.38115 (0.09) | 2.21906 (0.07) |
| **Arch**  **Aves**  **forelimb** | **BM1** | -7.211246 | 0.1565 | - | - | 0.00250 | - | 2.26927 (0.07) | - |
|  | ****BMS*** | *-8.211091* | *0.2580* | *-* | *-* | *0.00302* | *0.00165* | *2.26904 (0.08)* | *-* |
|  | **OU1** | -8.054088 | 0.2386 | 0.01104 | - | 0.00320 | - | 2.30751 (0.07) | - |
|  | **OUM** | -5.663262 | 0.0721 | 0.01049 | - | 0.00316 | - | 2.30983 (0.07) | 2.24573(0.16) |
|  | **OUMV** | -7.570373 | 0.1873 | 0.01358 | - | 0.00424 | 0.00208 | 2.32348 (0.08) | 2.23849(0.12) |
|  | **OUMA** | -4.090828 | 0.0328 | 0.00884 | 0.02416 | 0.00313 | - | 2.30341 (0.07) | 2.23403 (0.11) |
|  | **OUMVA** | -5.094451 | 0.0543 | 0.01669 | 0.01367 | 0.00460 | 0.00216 | 2.33728 (0.08) | 2.23536(0.13) |
|  |  |  |  |  |  |  |  |  |  |
| **Godefroit**  **Paraves**  **femur** | **BM1** | 50.86378 | 0.0021 | - | - | 0.00441 | - | 1.92804 (0.10) | - |
|  | **BMS** | 53.03376 | 0.0007 | - | - | 0.00438 | 0.00447 | 1.92927 (0.10) | - |
|  | **OU1** | 42.21795 | 0.1629 | 0.02875 | - | 0.00791 | - | 2.03064 (0.09) | - |
|  | **OUM** | 44.97324 | 0.0410 | 0.02169 | - | 0.00689 | - | 2.04556 (0.11) | 1.83516(0.14) |
|  | **OUMV** | 47.27163 | 0.0130 | 0.02152 | - | 0.00674 | 0.00689 | 2.04690 (0.11) | 1.83201(0.14) |
|  | **OUMA** | 48.75123 | 0.0062 | 0.03500 | 0.03360 | 0.01000 | - | 2.08054 (0.11) | 1.91152(0.13) |
|  | ****OUMVA*** | *39.10257* | *0.7737* | *1.00e-12* | *0.01434* | *0.00281* | *0.00501* | *2.20003 (0.10)* | *1.67139 (0.11)* |
| **Godefroit Paraves**  **forelimb** | **Model** | **AICc** | **ΔAICc** | **α 1** | **α 2** | **σ 1** | **σ 2** | **θ 1** | **θ 2** |
|  | **BM1** | 21.4447 | 0.5913 | - | - | 0.00355 | - | 2.20112 (0.10) | - |
|  | **BMS** | 23.66912 | 0.1944 | - | - | 0.00377 | 0.00338 | 2.19734 (0.10) | - |
|  | ****OU1*** | *23.55217* | *0.2061* | *0.00366* | *-* | *0.00395* | *-* | *2.21400 (0.10)* | *-* |
|  | **OUM** | 34.03737 | 0.0010 | 3.41795 | - | 0.68496 | - | 2.56090 (0.07) | 2.30470(0.06) |
|  | **OUMV** | 30.47394 | 0.0064 | 3.41795 | - | 1.07837 | 0.38760 | 2.56075 (0.08) | 2.30404 (0.04) |
|  | **OUMA** | 35.74764 | 0.0004 | 3.35099 | 3.33453 | 0.67257 | - | 2.57718 (0.07) | 2.30390(0.05) |
|  | **OUMVA** | NA | NA | NA | NA | NA | NA | NA | NA |

**Table S11.** A model of a split in the Aves, Paraves, and other for various OUwie models (BMS, OU1, OUM, OUMV, OUMA, OUMVA) for body size (femur length) and forelimb length). The best-fitting models for femur length in this three-split model (OUMVA, AICc = -13.21) are poorer-fitting compared to the best-fitting two-split (Paraves and remaining taxa) model (OUMVA, AICc = -16.15). The best-fitting model for forelimb length in the three-split model (OU1, AICc = -8.07) is equivalent to the best-fitting model in the two-split model (OU1, AICc = -13.21) (this result is when compared against equivalent models, BMS, OU1, OUM, OUMV, as the OUMA and OUMVA gave unreliable estimates in the three-split forelimb model).

|  | **Model** | **AICc** | **α 1** | **α 2** | **α 3** | **σ 1** | **σ2** | **σ3** | **θ1** | **θ2** | **θ3** |
| --- | --- | --- | --- | --- | --- | --- | --- | --- | --- | --- | --- |
| **Body Size (femur)** | **BM1** | 2.899546 | - | - | - | 0.0026 | - | - | 1.96861 (0.07) | - | - |
|  | **BMS** | 2.686201 | - | - | - | 0.0026 | 0.0017 | 0.0034 | 1.96861 (0.07) | - | - |
|  | **OU1** | -8.649253 | 0.0205 | - | - | 0.0039 | - | - | 2.02703 (0.06) | - | - |
|  | **OUM** | -4.908273 | 0.0186 | - | - | 0.0037 | - | - | 2.06757 (0.07) | 1.56675 (0.16) | 1.97809  (0.11) |
|  | **OUMV** | -5.915126 | 0.0209 | - | - | 0.0045 | 0.0023 | 0.0046 | 2.07687 (0.08) | 1.59579 (0.13) | 1.97736 (0.11) |
|  | **OUMA** | -11.03625 | 9e-14 | 0.0154 | 0.0162 | 0.0025 | - | - | 2.17405 (0.09) | 1.60578 (0.155) | 1.76970 (0.09) |
|  | **OUMVA** | -13.21522 | 9e-14 | 0.0213 | 0.0204 | 0.0020 | 0.0020 | 0.004 | 2.17405 (0.08) | 1.61945 (0.131) | 1.78599 (0.09) |
|  |  |  |  |  |  |  |  |  |  |  |  |
| **Forelimb Length** | **BM1** | -7.782006 | - | - | - | 0.0025 | - | - | 2.26831 (0.07) | - | - |
|  | **BMS** | -6.579632 | - | - | - | 0.0027 | 0.0017 | 0.0036 | 2.27689 (0.08) | - | - |
|  | **OU1** | -8.075751 | 0.0103 | - | - | 0.0031 | - | - | 2.30527 (0.07) | - | - |
|  | **OUM** | -3.421329 | 0.0096 | - | - | 0.0031 | - | - | 2.31942 (0.08) | 2.18964 (0.19) | 2.27275 (0.14) |
|  | **OUMV** | -2.314517 | 0.0164 | - | - | 0.0049 | 0.0022 | 0.0041 | 2.34416 (0.09) | 2.19660 (0.14) | 2.29444 (0.14) |
|  | **OUMA** | NA | NA | NA | NA | NA | NA | NA | NA | NA | NA |
|  | **OUMVA** | NA | NA | NA | NA | NA | NA | NA | NA | NA | NA |

**Allometry of femur and forelimb evolution**

**Table S12.** PGLS models to clarify that differences between the main phylogeny and Godefroit et al (2013) phylogeny are not due to sample size (a), and a lot of the differences can be explained by reclassification of just two taxa (*Anchiornis* and *Xiaotingia*) when phylogenies are reduced to the same size (b). The regression coefficients, estimates of Lambda, R squared, and AICc of all models are shown. In the Godefroit phylogeny a shallower Aves slope is seen compared to the main phylogeny when both phylogenies are reduced to the same number of taxa (a). These patterns are reversed when *Anchiornis* and *Xiaotingia* are classified as Aves (in the main phylogeny) or Paraves (in the Godefroit phylogeny) (b).

| *(a)* | **Main Tree** | | | **Godefroit *et al.* 2013 tree** | | |
| --- | --- | --- | --- | --- | --- | --- |
|  | Regression coeff (± s.e.) | t | *p* | Regression coeff (± s.e.) | **t** | ***p*** |
| **Intercept** | 0.454 (0.377) | 1.20 | 0.236 | **1.197 (0.262)** | **4.56** | **<0.001** |
| **Femur (avian slope)** | **1.07 (0.226)** | **4.74** | **<0.001** | **0.606 (0.149)** | **4.06** | **<0.001** |
| **Paraves (intercept term)** | 0.585 (0.412) | 1.41 | 0.164 | -0.328 (0.324) | -1.01 | 0.31 |
| **Theropods (intercept term)** | -0.536 (0.417) | -1.28 | 0.207 | **-1.256 (0.319)** | **-3.93** | **<0.001** |
| **Paraves (slope)** | -0.419 (0.244) | -1.71 | 0.09 | 0.134 (0.181) | 0.73 | 0.46 |
| **Theropods (slope)** | 0.053 (0.239) | 0.22 | 0.82 | **0.510 (0.170)** | **3.00** | **<0.01** |
| **Lambda** | **0.477** | | | **0.356** |  |  |
| **R Squared** | **0.8839** | | | **0.8728** |  |  |
| **AICc** | **-66.38711** | | | **-62.75699** |  |  |
| *(b)* | **Reclassified Main Tree** | | | **Reclassified Godefroit Tree** | | |
|  | Regression coeff (± s.e.) | t | *p* | Regression coeff  (± s.e.) | **t** | ***p*** |
|  |  |  |  |  |  |  |
| **Intercept** | **1.118 (0.272)** | **4.10** | **<0.001** | 0.400 (0.392) | 1.02 | 0.313 |
| **Femur (avian slope)** | **0.647 (0.155)** | **4.17** | **<0.001** | **1.100 (0.235)** | **4.67** | **<0.001** |
| **Paraves (intercept term)** | -0.221 (0.327) | -0.06 | 0.503 | 0.654 (0.424) | 1.54 | 0.132 |
| **Theropods (intercept term)** | **-1.200 (0.328)** | **-3.65** | **<0.001** | -0.462 (0.430) | -1.07 | 0.282 |
| **Paraves (slope)** | 0.078 (0.185) | 0.42 | 0.674 | -0.452 (0.252) | -1.79 | 0.081 |
| **Theropods (slope)** | **0.482 (0.17)** | **2.75** | **<0.01** | 0.016 (0.248) | 0.06 | 0.945 |
| **R Squared** | **0.8771** | | | **0.8804** |  |  |
| **Lambda** | **0.549** | | | **0.318** |  |  |
| **AICc** | **-64.88075** | | | **-64.64087** |  |  |

**Table S13.** Use of alternative data sources for body size and forelimb length (Dececchi and Larrson 2013). Snout-ventral length (SVL) and an alternative forelimb data show similar patterns to the femur data and forelimb length used in the main analysis. The main difference between the main tree and Godefroit tree is the shallower slope in the Godefroit tree for the Aves (as seen in the main analysis). When *Anchiornis* and *Xiaotingia* are reclassified (as Aves in the main phylogeny, and Paraves in the Godefroit phylogeny) a shallower Aves slope is seen in the main phylogeny, and a steeper slope in the Godefroit phylogeny; these are similar results to the main dataset (supplementary table 9). However, it should also be noted these differences are not as pronounced as they are with the main dataset.

|  | **Full main tree** | | | **Godefroit tree** | | |
| --- | --- | --- | --- | --- | --- | --- |
|  | Regression coeff (± s.e.) | t | *p* | Regression coeff (± s.e.) | **t** | ***p*** |
| **Intercept** | 0.062 (0.398) | 0.15 | 0.875 | **1.444 (0.635)** | **2.27** | **0.03** |
| **SVL (avian slope)** | **0.910 (0.173)** | **5.23** | **<0.001** | 0.310 (0.262) | 1.18 | 0.24 |
| **Paraves (intercept term)** | 0.178 (0.460) | 0.38 | 0.700 | -1.338 (0.684) | -1.95 | 0.06 |
| **Theropods (intercept term)** | **-1.198 (0.455)** | **-2.63** | **0.011** | **-2.48 (0.685)** | **-3.62** | **<0.01** |
| **Paraves (slope)** | -0.124 (0.196) | -0.63 | 0.532 | 0.513 (0.282) | 1.81 | 0.08 |
| **Theropods (slope)** | 0.286 (1.5196) | 1.51 | 0.136 | **0.853 (0.276)** | **3.09** | **0.004** |
| **R Squared** | 0.9184 | | | 0.901 |  |  |
| **Lambda** | 0 | | | 0.656 |  |  |
| **AICc** | -80.59581 | | | -61.00815 |  |  |
|  | **Reclassified Main Tree** | | | **Reclassified Godefroit Tree** | | |
|  | Regression coeff (± s.e.) | t | *p* | Regression coeff (± s.e.) | **t** | ***p*** |
| **Intercept** | 0.266 (0.367) | 0.72 | 0.473 | 1.149 (0.902) | 1.27 | 0.21 |
| **SVL (avian slope)** | **0.800 (0.153)** | **5.22** | **<0.001** | 0.429 (0.383) | 1.12 | 0.27 |
| **Paraves (intercept term)** | 0.036 (0.437) | 0.08 | 0.934 | -0.917 (0.942) | -0.97 | 0.38 |
| **Theropods (intercept term)** | **-1.353 (0.445)** | **-3.03** | **<0.001** | **-2.224 (0.943)** | **-2.35** | **0.02** |
| **Paraves (slope)** | -0.057 (0.180) | -0.31 | 0.750 | 0.357 (0.399) | 0.89 | 0.37 |
| **Theropods (slope)** | **0.381 (0.175)** | **2.17** | **0.035** | 0.745 (0.394) | 1.88 | 0.06 |
| **R Squared** | 0.8843 | | | 0.8856 |  |  |
| **Lambda** | 0.498 | | | 0.420 |  |  |
| **AICc** | -83.09681 | | | -54.04607 |  |  |

**Table S14.** A PGLS model of 3 regimes (Aves, other Paraves, and other taxa) with interactions between femur and forelimb length is the best-fitting model. Best-fitting ANOVA models for forelimb on femur length when the phylogeny is split into Paraves, Aves, and Paraves (‘Three’), Paraves and remaining taxa (‘Paraves’), and Aves and remaining taxa (‘Aves’). The interaction term ‘*’ allows for different slopes and intercepts and ‘+’ for different intercepts only. In all trees the best-fitting model allows for different slopes and intercepts in the model with three groups (‘Three’).

|  | Main | Godefroit | Main tree reduced to *Aurornis* size |
| --- | --- | --- | --- |
| Femur*Three | **-138.0209** | **-62.75699** | **-66.38711** |
| Femur+Three | -126.4931 | -54.99994 | -57.82168 |
| Femur*Paraves | -127.0633 | -62.3636 | -64.19396 |
| Femur+Paraves | -123.6223 | -53.04339 | -56.10628 |
| Femur*Aves | -112.4896 | -47.31856 | -47.18103 |
| Femur+Aves | -111.222 | -47.8828 | -48.8421 |

**Supplementary References**

Agnolin F.L., J.E. Powell, F.E. Novas, M. Kundrat. 2012 New alvarezsaurid Dinosauria, Theropoda from uppermost Cretaceous of north-western Patagonia with associated eggs. Cret. Res. 35:33-36.

Burnham D.A., A. Feduccia, L.D. Martin, and A.R. Falk. 2011. Tree climbing - a fundamental avian adaptation. J. Syst. Palaeontol. 9:103-107.

Butler, R. J., and P. Upchurch. 2007. Highly incomplete taxa and the phylogenetic relationships of the theropod dinosaur Juravenator starki. J. Vert. Paleontol. 27:253-256.

Carrano, M. T. 2006. 8 Body-Size Evolution in the Dinosauria. Amniote paleobiology: perspectives on the evolution of mammals, birds, and reptiles. 225.

Clarke J.A., Z.H. Zhou, and F.C. Zhang. 2006. Insight into the evolution of avian flight from a new clade of Early Cretaceous ornithurines from China and the morphology of *Yixianornis* *grabaui*. J. Anat. 208:287-308.

Csiki Z, M. Vremir, S.L. Brusatte, and M.A. Norell. 2010 An aberrant island-dwelling theropod dinosaur from the Late Cretaceous of Romania. Proc. Natl. Acad. Sci. USA. 107:15357-15361.

Godefroit, P., H. Demuynck, G. Dyke, D. Hu, F. Escuillié, and P. Claeys. 2013b. Reduced plumage and flight ability of a new Jurassic paravian theropod from China. Nat. Commun. 4:1394.

Gong E-P., L.D. Martin, D.A. Burnham, A.R. Falk, and L-H. Hou. 2012 A new species of Microraptor from the Jehol Biota of northeastern China. Palaeoworld. 21:81-91

Hu D., L. Hou, and X. Xu. 2009 A new enantiornithine bird from the Lower Cretaceous of western Liaoning, China. J. Vert. Paleontol. 29:154-161.

Liyong, J., C. Jun, and P. Godefroit. 2012. A new basal ornithomimosaur (Dinosauria: theropoda) from the Early Cretaceous Yixian Formation, Northeast China. Bernissart Dinosaurs and Early Cretaceous Terrestrial Ecosystems. 467-487.

Lloyd, G.T., K. E. Davis, D. Pisani, J. E. Tarver, M. Ruta, M. Sakamoto, D.W.E. Hone, R. Jennings, and M. J. Benton. Dinosaurs and the Cretaceous terrestrial revolution. 2008. Proc. R. Soc. B. 275:2483-2490.

Longrich N. 2009. An ornithurine-dominated avifauna from the Belly River Group Campanian, Upper Cretaceous of Alberta, Canada. Cret. Res. 30:161-177.

Longrich N.R., P.J. Currie, and Z-M. Dong. 2010. A new oviraptorid (Dinosauria: Theropoda) from the Upper Cretaceous of Bayan Mandahu, Inner Mongolia. Palaeontology. 53:945-960.

Novas, FE, and D. Pol. 2005. New evidence on deinonychosaurian dinosaurs from the Late Cretaceous of Patagonia. Nature. 433:858-861.

O'Connor, J.K., and Z. Zhou. 2013. A redescription of Chaoyangia beishanensis (aves) and a comprehensive phylogeny of mesozoic birds. J. Syst. Palaeont. 11:889-906.

Senter, P. 2007 A new look at the phylogeny of Coelurosauria Dinosauria: Theropoda. J. Syst. Palaeontol. 5:429-463.

Senter P, J.I Kirkland, J. Bird, and J.A. Bartlett. 2010. A new troodontid theropod dinosaur from the Lower Cretaceous of Utah. Plos One. 5(12) e14329.

Senter P, J.I Kirkland, and D.D. DeBlieux. 2012a Martharaptor greenriverensis, a new theropod dinosaur from the Lower Cretaceous of Utah. PloS One. 7(8):e43911

Senter P., J.I. Kirkland, D.D. DeBlieux, S. Madsen, and N. Toth. 2012b. New dromaeosaurids (Dinosauria: Theropoda) from the Lower Cretaceous of Utah, and the evolution of the dromaeosaurid tail. Plos One. 7(5). e36790.

Turner., A.H., P.J. Makovicky, and M.A. Norell. 2012 A review of dromaeosaurid systematics and paravian phylogeny. Bull. Am. Mus. Nat. Hist. 371:1-206.

Wang X, and Z. Zhang. 2011 Enantiornithine birds in China. Acta Geol. Sin.-Engl. 85:1211-1223.

Wang, X., G. Dyke, P. Godefroit. 2013 A new specimen of *Jeholornis*-like long-tailed bird shows that *Jixiangornis* is a junior synonym of *Jeholornis* *prima*. Acta. Palaeontol. Pol. Online early.

Xu, X., Z. Zhou, X. Wang, X. Kuang, F. Zhang, and X. Du. 2003. Four-winged dinosaurs from China. Nature.  6921:335-340.

Xu, X and X.L. Wang. 2004. A new troodontid (Theropoda: Troodontidae) from the Lower Cretaceous Yixian Formation of western Liaoning, China. Acta Geol. Sin.-Engl. 78:22-26.

Xu X, J.N. Choiniere, M.D. Pittman, Q. Tan, D. Xiao, Z. Li, L. Tan, J.M. Clark, M.A. Norrell, D.W.E. Hone *et al.* 2010. A new dromaeosaurid Dinosauria: Theropoda from the Upper Cretaceous Wulansuhai Formation of Inner Mongolia, China. Zootaxa 2403:1-9.

Xu X., Q. Tan, C. Sullivan, F. Han, and D. Xiao. 2011 A short-armed troodontid dinosaur from the Upper Cretaceous of Inner Mongolia and its implications for troodontid evolution. Plos One. 6(9). e22916.

Zanno L.E., D.J. Varricchio, P.M. O'Connor, A.L. Titus, and M.J. Knell. 2011. A new troodontid theropod, *Talos* *sampsoni* gen. et sp. nov., from the Upper Cretaceous Western Interior Basin of North America. Plos One. 6(9) e24487.

Zanno LE, D.D. Gillette, L.B. Albright, and A.L. Titus. 2009 A new North American therizinosaurid and the role of herbivory in 'predatory' dinosaur evolution. Proc. R. Soc. B. 276:3505-3511.

Zhang F.C., Z.G. Zhou, X. Xu, and X.L. Wang. 2002 A juvenile coelurosaurian theropod from China indicates arboreal habits. Naturwissenschaften. 89:394-398.

Zhang FC, and Z.H. Zhou. 2000. A primitive enantiornithine bird and the origin of feathers. Science. 290:1955-1959.

Zhang, Z., C. Gao, Q. Meng, J. Liu, L. Hou, and G. Zheng. 2009. Diversification in an Early Cretaceous avian genus: evidence from a new species of *Confuciusornis* from China. J. Ornithol. 150:783-790.
